# Glia cell-derived extracellular vesicles as modulators in spinal cord injury repair

**DOI:** 10.1038/s41393-026-01231-z

**Published:** 2026-06-09

**Authors:** Flavia Millesi, Elena Millesi, Maximilian Härtinger, Sophie Steinwenter, Alexander Rührnößl, Aida Naghilou, Christine Radtke

**Affiliations:** 1 Department of Plastic, Reconstructive and Aesthetic Surgery, Medical University of Vienna, 1090 Vienna, Austria; 2 Austrian Cluster for Tissue Regeneration, 1200 Vienna, Austria; 3 Division of Plastic and Reconstructive Surgery, Mayo Clinic, 55905 Rochester, MN USA

**Keywords:** Spinal cord injury, Molecular neuroscience

## Abstract

**Study design:**

Narrative review.

**Objectives:**

The aim of this review was to summarize and critically evaluate current evidence on glial cell–derived extracellular vesicles (EVs) as therapeutic mediators in spinal cord injury (SCI), with a focus on their cell-specific functions and phase-dependent effects.

**Methods:**

We narratively synthesized preclinical in vitro and in vivo studies investigating EVs derived from astrocytes, microglia, oligodendrocytes, Schwann cells, and olfactory ensheathing cells in the context of spinal cord injury and related central nervous system pathologies.

**Results:**

Glial cell–derived EVs exhibit diverse and cell-type–specific effects following SCI. Astrocyte-derived EVs (ADEVs) contain neuroprotective proteins and microRNAs that regulate inflammation and support neural repair. Microglia-derived EVs (MGEVs) display dual roles, with pro-inflammatory EVs exacerbating secondary injury, while anti-inflammatory EVs promote recovery. Oligodendrocyte-derived EVs (ODEVs) contribute to metabolic support and remyelination but may also carry inhibitory molecules that limit axonal regeneration. Schwann cell-derived EVs (SCEVs) reduce scar formation and enhance axonal growth, in some models outperforming Schwann cell transplantation. Olfactory ensheathing cell-derived EVs (OECEVs) promote axonal regeneration, likely through modulation of the extracellular environment and enhanced debris clearance. Across injury phases, glial EVs may protect the blood–brain barrier in the acute stage, modulate inflammation and angiogenesis in the subacute stage, and support axonal regrowth, remyelination, and synaptic remodeling in the chronic stage.

**Conclusions:**

Glial cell–derived extracellular vesicles represent a promising, cell-free therapeutic strategy for SCI. While preclinical evidence highlights substantial regenerative and immunomodulatory potential, challenges remain regarding EV isolation, targeting, and delivery. Addressing these limitations will be essential for advancing clinical translation.

## Introduction

Spinal cord injury (SCI) is a devastating neurological condition leading to persistent motor, sensory, and autonomic impairments with profound physical, psychological, and socioeconomic consequences. Traumatic events are the predominant cause for SCI and include road traffic accidents, falls, sports injuries, and acts of violence. Non-traumatic causes, such as infections, tumors, vascular injuries, and degenerative diseases, account for a smaller but clinically relevant proportion of cases [1].

**Fig. 1:**
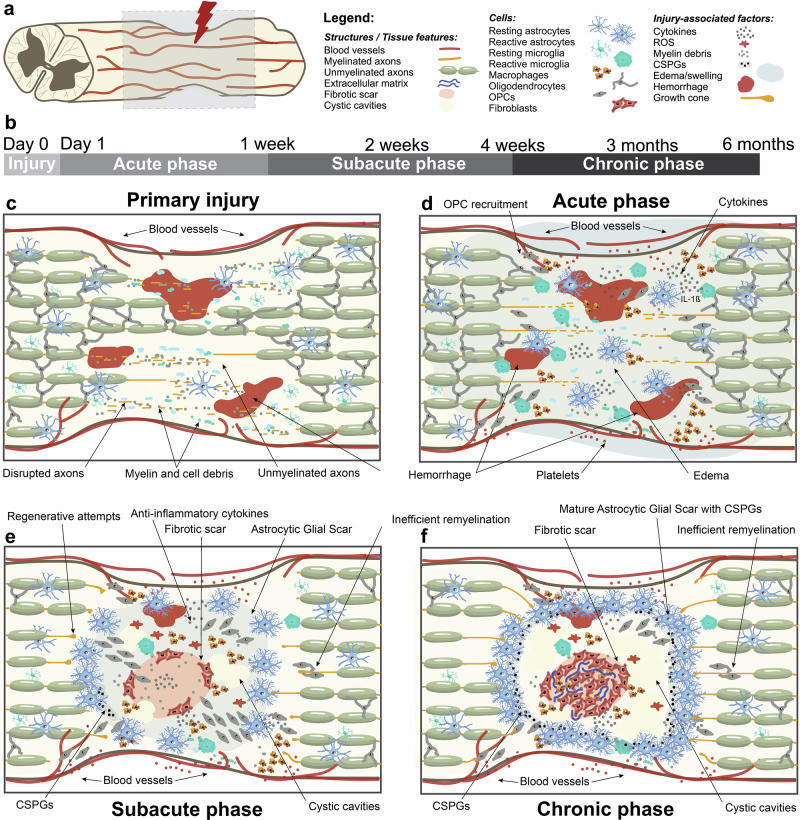
Spinal cord injury phases. **a** Schematic representation of the spinal cord indicating the site of injury. **b** Timeline after injury. Schematic illustrations depict the morphological changes within the spinal cord at (**c**) immediately after injury, (**d**) during the acute phase, (**e**) the sub-acute phase, and (**f**) the chronic phase.

Mechanistically, SCI can vary significantly in terms of injury type and severity, ranging from contusions and partial lacerations to complete transections of the spinal cord. The pathophysiology is commonly divided into primary and secondary injury phases [1]. Primary injuries refer to the initial mechanical insult of the spinal cord. In contrast, secondary injury comprises a cascade of pathological processes triggered by the primary trauma, including vascular disruption, ischemia, excitotoxicity, oxidative stress, inflammation, and cell death. Following primary injury, SCI progresses through distinct phases: acute, subacute, and chronic (Fig. 1) [1]. The acute phase occurs within minutes to hours post-injury and involves immediate tissue damage, hemorrhage, and disruption of spinal cord vasculature. The subacute phase, spanning days to weeks, involves inflammatory responses, apoptotic and necrotic cell death, progressive tissue remodeling, and the development of glial and fibrotic scarring. Finally, the chronic phase, which may persist for weeks to months or longer, is characterized by scar maturation, axonal degeneration, cystic cavitation, and limited neuroplastic adaptations. Understanding these phases is crucial for developing effective therapeutic interventions [Supplementary references 1–4].

Based on the extent and location of the injury, the consequences of SCIs may include motor and sensory deficits and autonomic dysfunctions [1]. Injury severity is clinically classified according to the ASIA/ISCoS International Standards for Neurological Classification of Spinal Cord Injury (ISNCSCI), which includes assessment of motor and sensory function and assigns an American Spinal Injury Association Impairment Scale (AIS) grade ranging from AIS A (complete injury) to AIS E (preserved motor and sensory function on examination). Beyond neurological impairment, SCI has major psychosocial consequences [2]. Motor and sensory deficits can severely limit mobility and independence. Autonomic dysfunction, including autonomic dysreflexia and orthostatic hypotension, further complicates daily living and social participation. In addition, individuals with SCI are at increased risk of psychological comorbidities such as depression and anxiety. Because SCI often necessitates long-term medical care, rehabilitation, and social support, it also places a substantial burden on families and healthcare systems worldwide [Supplementary references 5–12].

## Current treatment strategies for spinal cord injuries

Current treatment approaches for SCI are multifaceted to address both the primary injury and secondary complications [3]. In general, therapeutic approaches can be differentiated between acute and long-term management. In the acute phase, the primary goals are to stabilize the patient, preserve spinal cord perfusion, and limit further neurological deterioration through surgical and medical interventions. Decompression surgery allows to relieve pressure off the spinal cord and, when indicated, improve outcomes by restoring perfusion and reducing ongoing. Acute medical management may also include hemodynamic support to maintain adequate blood pressure and spinal cord perfusion, as well as the prevention and treatment of complications such as infection. While several neuroprotective pharmacological approaches have been explored, their clinical benefit remains limited, and the use of agents such as high-dose corticosteroids remains controversial due to inconsistent evidence of neurological improvement and an increased risk of complications such as infection, gastrointestinal bleeding, and sepsis [Supplementary references 13–17].

The subacute phase, which extends from hours to weeks post-injury, is characterized by a worsening of secondary injury mechanisms, including neuroinflammation, apoptosis, demyelination, and scar formation [3]. The glial response in this phase is complex: while reactive astrocytes and associated scar-forming cells help contain tissue damage and restrict the spread of inflammation early after injury, persistent scar tissue later becomes a major barrier to axonal regeneration. As SCI progresses into the chronic phase, long-term structural and functional impairments become more established. Axonal degeneration continues, cystic cavitation may develop, and inhibitory molecules within scar tissue and damaged myelin further reduce the regenerative potential of the injured spinal cord. Long-term management focuses on rehabilitation, complication prevention, and improvement of functional recovery and quality of life. Physical and occupational therapy is crucial to maximize functional recovery and adapt to altered mobility in order to adjust to daily activities and promote self-care independence. Furthermore, neuropathic pain and spasticity are common challenges faced by SCI patients. To improve function further, neuromodulatory and assistive strategies have increasingly been explored. Techniques such as trans-spinal direct current stimulation and trans-spinal pulsed current stimulation show promise in improving sensorimotor and autonomic functions [3]. Moreover, emerging technologies such as brain-machine interfaces and robotic exoskeletons are investigated to enhance mobility and independence [Supplementary references 18–28].

Despite these advances, the regenerative capacity of the spinal cord remains limited. This is due to multiple factors, including persistent neuroinflammation, the inhibitory nature of glial and fibrotic scar tissue, the persistence of myelin-associated inhibitors, and the lack of intrinsic regenerative mechanisms in CNS neurons. Among biological strategies, cell transplantation has emerged as a major therapeutic avenue [4], with preclinical and early clinical studies exploring the use of Schwann cells (SCs), olfactory ensheathing cells (OECs), oligodendrocyte progenitor cells (OPCs), neural stem/progenitor cells, and mesenchymal stem cells (MSCs). These approaches aim to provide trophic support, promote remyelination, and offer structural guidance for regenerating axons. However, transplantation faces significant limitations, including poor long-term survival and integration of grafted cells, concerns regarding immune compatibility and phenotypic stability, invasive delivery requirements, and present challenges in standardization and large-scale production.

Extracellular vesicles (EVs) offer a promising cell-free alternative that may circumvent many of these barriers [5]. By transferring bioactive cargo such as proteins, lipids, and RNAs, EVs recapitulate many of the beneficial paracrine effects of transplanted cells without the associated risks of engraftment or uncontrolled proliferation. EVs also exhibit lower immunogenicity, greater stability during storage and transport, and enhanced ability to cross biological barriers, such as the blood-spinal cord barrier. Recent preclinical studies demonstrate that glia-derived EVs can modulate inflammation, promote remyelination, and support neuronal survival, thereby positioning them as a next-generation therapeutic modality.

In this narrative review, we highlight current research on glial cell-derived EVs in SCI and discuss their potential to advance neuroprotection and regeneration after injury. The included studies were identified using the PubMed database. Search terms included combinations of “extracellular vesicles”, “exosomes”, “spinal cord injury”, and either “astrocytes”, “microglia”, “oligodendrocytes”, “Schwann cells”, “olfactory ensheathing cells”, “ependymal cells”, or “satellite glial cells”. The search covered publications up to the end of 2025, with a focus on recent studies and mechanistic investigations. Original research articles were included based on their relevance to glial cell-derived EVs in the context of spinal cord injury and CNS repair. Review articles were excluded from the primary analysis but screened for additional references. Studies from related CNS or peripheral nervous system models were included to support mechanistic interpretation. To further contextualize the evidence, a structured appraisal of SCI-specific in vivo studies was performed based on key methodological domains adapted from CRIME-Q [6], including injury model, timing of intervention, EV characterization, and reporting of randomization and blinding (Supplementary table 1). While most studies employed established rodent SCI models and demonstrated acceptable to strong EV characterization, substantial heterogeneity was observed in injury paradigms, delivery routes, and dosing strategies. In addition, reporting of bias control measures such as randomization, blinding, and sample size justification was inconsistent across studies. These factors limit direct comparability and should be considered when interpreting the translational potential of glia-derived EVs.

## Therapeutic approaches with extracellular vesicles

The intercellular communication via cell-derived membrane vesicles is highly conserved and employed throughout multiple kingdoms of life [7]. As such, these vesicles have had various names since their respective discoveries. In order to accommodate all membrane-delimited vesicles of cellular origin in the nano- to micrometer size range that cannot replicate, the current nomenclature recommends the use of the term EV, or non-vesicular extracellular particle when the vesicular nature cannot be confirmed. In recent years, the involvement of EVs in a plethora of physiological processes has been revealed, ranging from developmental processes and tissue homeostasis to regeneration and repair processes [Supplementary references 29–31]. However, EVs have also been shown to mediate pathophysiological processes, such as cancer progression, metastatic niche formation, neurodegenerative disease propagation and (neuro-)inflammatory processes [7]. To influence cellular behavior, EVs may interact with target cells either by surface receptor activation followed by signaling, uptake and unpacking, or membrane fusion. Different subsets of EVs are formed by outward budding of the cell membrane or inward budding into the multivesicular endosome which subsequently fuses with the cell membrane and releases these vesicles. Owing to their biogenesis, EVs often reflect the molecular characteristics of their cell of origin, including membrane-associated proteins and luminal cargo, which makes them attractive candidates for both diagnostic and therapeutic applications [Supplementary references 32,33]. Fusco et al. [8] have recently reviewed the literature on EVs as therapeutic agents including first in human clinical trials. The majority of studies used EVs enriched from MSCs, often administered locally, for example via EV-loaded hydrogels to support tissue repair. Cell culture-based EV production offers important advantages, including controlled generation conditions, reproducible isolation workflows, and the possibility of genetically modifying the source cells to tailor EV cargo. While MSC-derived EVs offer promising characteristics, including immunomodulatory effects and scalability, they may lack tissue-specific signaling relevant to the injured central nervous system. Alternative strategies therefore focus on EVs that are native to, or actively involved in, the injured tissue. In the context of SCI, glial cell-derived EVs may be especially relevant because they reflect endogenous repair-associated signaling pathways of the nervous system. Their cargo is closely linked to key processes in SCI pathology and repair, including neuroinflammation, myelin turnover, axonal guidance, and scar formation. Rather than introducing entirely new biological functions, these vesicles may enhance or redirect intrinsic regenerative and immunomodulatory responses already initiated by glial cells after injury. At the same time, because EV cargo depends strongly on the activation state of the parental cell, glial EVs may also exert detrimental effects, underscoring the need for a cell-type-specific and context-dependent evaluation [Supplementary references 34–42].

## Glia cell-derived EVs for spinal cord injury

### Astrocyte-derived EVs

Astrocytes are the most abundant glial cells in the mammalian CNS. They help form and maintain the blood-brain barrier, regulate blood flow, provide metabolic support, recycle neurotransmitters, and modulate immune signaling and synaptic transmission, ensuring neural network stability and efficiency [9]. In response to SCI, astrocytes become reactive, undergoing morphological and functional changes characterized by hypertrophy and the upregulation of glial fibrillary acidic protein (GFAP). These reactive astrocytes form a glial scar, which serves to isolate the injured area but can also inhibit axonal regrowth. Additionally, astrocytes are deeply involved in the process of remyelination following demyelinating insults. They release growth factors, cytokines, and chemokines that promote the proliferation and differentiation of oligodendrocyte precursor cells (OPCs), while also secreting factors that can either support or inhibit oligodendrocyte maturation [9].

Recent SCI-specific studies show that the effects of ADEVs depend on the activation state of the parent astrocytes. EVs from quiescent astrocytes reduce microglial activation, decrease pro-inflammatory cytokines (e.g., TNFα, IL-6), attenuate neuronal apoptosis, and promote axonal growth and oligodendrocyte maturation, accompanied by increased GAP43, NF200, MBP, and MAG expression and a shift toward an anti-inflammatory microglial phenotype ( ↑ CD206, ↓iNOS/CD86), resulting in improved functional recovery after a traumatic contusion SCI in mice with treatment given in the acute-subacute stage [10]. In contrast, EVs from reactive astrocytes enhance microglial activation, increase neuronal apoptosis, and impair recovery. This divergence is linked to differences in EV cargo, particularly the enrichment of pro-inflammatory mediators such as CCL7 in reactive ADEVs. CCL7-containing EVs promote microglial recruitment and pro-inflammatory activation, whereas CCL7 knockdown reduces inflammation and neuronal apoptosis and improves functional outcomes in SCI models [10]. In addition to immune modulation, astrocyte-derived EVs have also been shown to directly influence vascular repair after SCI [11]. EVs derived from A2 astrocytes promote restoration of the blood-spinal cord barrier in an acute traumatic contusion SCI mouse model, as demonstrated by reduced Evans Blue leakage and increased expression of tight junction proteins such as ZO-1, occludin, and claudin-5. Mechanistically, these effects are mediated by miR-5121 enriched in A2 astrocyte-derived EVs, which activates autophagy in vascular endothelial cells, as indicated by increased LC3-II and Beclin-1 expression. Inhibition of miR-5121 abolishes these protective effects, demonstrating a causal role of EV cargo in regulating endothelial function and barrier integrity after SCI [11].

Beyond SCI, ADEVs have been shown to promote neuronal survival and repair in other CNS injury models. In traumatic brain injury, ADEVs reduced neuroinflammation, promoted the differentiation and migration of OPCs, and supported white matter repair. Similarily, ADEVs from hypoxic astrocytes improved BBB integrity through the miR-27a-3p/Wnt/β-catenin pathway in intracerebral hemorrhage models, and fibulin-2-containing ADEVs promoted synaptogenesis through TGF-β signaling. By enhancing synapse formation, ADEVs could potentially mitigate the loss of neuronal connections and support the re-establishment of functional neural circuits, thereby contributing to improved motor and sensory outcomes in SCI patients. In addition, ADEV-associated miRNAs such as miR-138, miR-29a and miR-143-3p regulate inflammatory pathways, including NF-κB/NLRP3 signaling and endothelial activation. ADEVs’ cargo of factors like BDNF and miRNAs targeting autophagy (e.g., miR-92b-3p) might protect spinal neurons and support axonal growth and plasticity. However, these findings are derived from non-SCI models and should be interpreted cautiously when extrapolated to spinal cord injury.

**Fig. 2:**
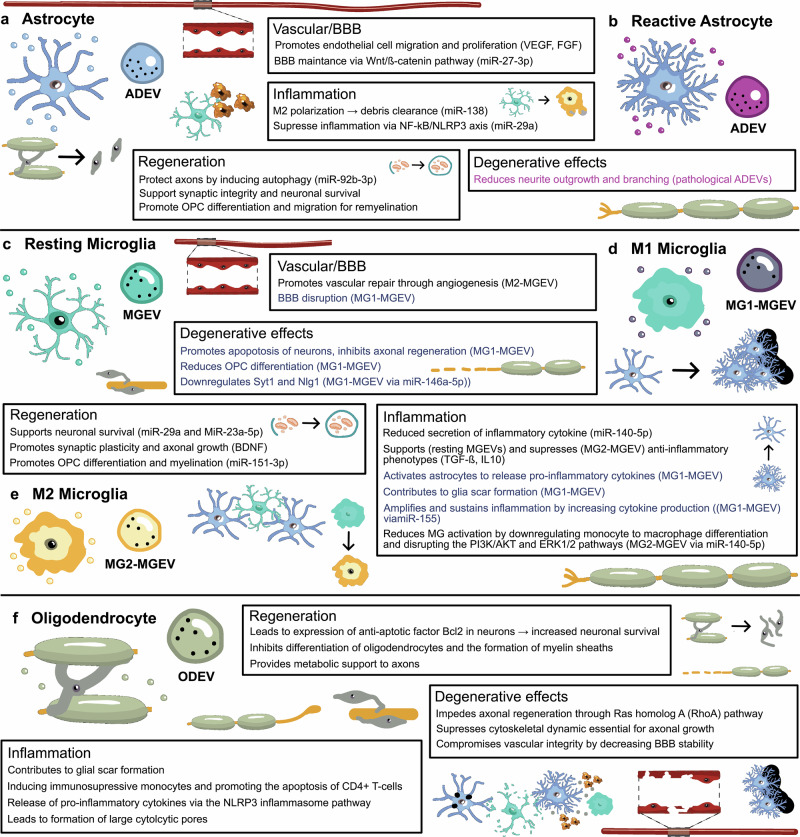
Astrocyte, Microglia and Oligodendrocyte-derived EVs and their effects. **a** Cargo and effects of EVs derived from astrocytes in resting state. **b** Cargo and effects of EVs derived from reactive astrocytes. **c** Cargo and effects of EVs derived from microglia in resting state. **d** Cargo and effects of EVs derived from reactive microglia in their M1 state. **e** Cargo and effects of EVs derived from reactive microglia in their M2 state. **f** Cargo and effects of EVs derived from oligodendrocytes.

With these multifaceted actions, ADEVs hold promise for reducing neuroinflammation, preserving structural integrity, and promoting functional recovery in SCI. ADEVs are known to carry neuroprotective proteins, such as heat shock proteins (HSPs) and apolipoproteins like ApoE, which support synaptic integrity and neuronal survival. Further, ADEVs influence vascular remodeling by carrying angiogenic factors such as VEGF and FGF-2, enhancing endothelial cell proliferation and migration to repair damaged vessels, which is vital for sustaining neuronal health and recovery after brain injury (Fig. 2a, Supplementary References 43–55). However, an important factor for the success of ADEVs is the culture of astrocytes prior to EV isolation: ADEVs derived from reactive astrocytes differ in size, protein composition, and cargo compared to those from quiescent astrocytes [Supplementary references 56–60]. Similarly, the EVs from reactive astrocytes have also been found to contain different bioactive substances compared to resting astrocytes (Fig. 2b). Reactive ADEVs are also taken up more efficiently by neurons compared to those from non-activated astrocytes, due to the enrichment of specific surface proteins on the EVs but exert detrimental effects, including reduced neurite outgrowth, impaired branching, and altered neuronal electrophysiological properties. In the context of SCI, this underscores that astrocyte-derived EVs can either promote repair or exacerbate injury, depending on astrocyte activation state and cargo composition.

Taken together, these findings indicate that astrocyte-derived EVs may exert phase-specific effects following SCI. In the acute phase, ADEVs appear particularly relevant for stabilizing the blood-spinal cord barrier and limiting early inflammatory responses, as demonstrated by their effects on endothelial integrity and cytokine modulation. In the subacute phase, ADEVs contribute to shaping the injury microenvironment by regulating microglial activation, promoting oligodendrocyte maturation, and supporting axonal growth. However, these effects are highly dependent on the activation state of the parent astrocytes, as EVs derived from reactive astrocytes can exacerbate inflammation and neuronal damage. Therefore, therapeutic application of ADEVs in SCI will likely require precise control over astrocyte activation state and timing of administration to maximize beneficial effects while avoiding detrimental signaling.

### Microglia-derived EVs

Microglia, the immune sentinels of the CNS, originate from erythromyeloid precursors and are unevenly distributed across the CNS. They play crucial roles in neurodevelopmental processes, including neural precursor cell proliferation, differentiation, synaptogenesis, and neuronal survival [4]. Their absence can negatively affect neuronal integrity, highlighting their importance in maintaining neural health. In response to SCI, microglia become reactive, altering their morphology and behavior to address the damage [4]. They extend processes to protect the injury site in acute cases and become amoeboid in more extensive damage. Microglia play a vital role in remyelination by phagocytosing myelin debris, secreting regenerative factors, and modulating the extracellular matrix (ECM). Receptors like CX3CR1 and RXR-γ are crucial for myelin debris clearance, with deficiencies leading to impaired clearance. Microglia secrete a range of pro-regenerative factors, including growth factors (IGF1, FGF2), cytokines (IL-1β, IL-4), and chemokines (CXCL12), which regulate OPC proliferation and differentiation [4].

Similar to ADEVs, recent SCI-specific studies demonstrate that the effects of microglia-derived EVs (MGEVs) is dual. EVs derived from anti-inflammatory or M2-like microglia promote neuronal survival, reduce apoptosis, and improve functional recovery after SCI. For example, MGEVs carrying miR-151-3p, injected for three consecutive days after a contusion injury to the murine SCI, have been shown to attenuate neuronal apoptosis by regulating the p53/p21/CDK1 signaling pathway, thereby supporting axonal regeneration and improving neurological outcomes [12]. In addition, systemic injection of MGEVs has been shown to reduce oxidative stress in an acute rat contusion model of SCI by decreasing reactive oxygen species levels and downregulating NOX2 expression, which promotes endothelial cell survival and supports vascular integrity [13]. Beyond direct neuroprotection, MGEVs also contribute to intercellular crosstalk within the injured spinal cord. EVs derived from M2 microglia suppressed A1 astrocyte activation, thereby reducing neurotoxic astrocyte responses and promoting a more permissive environment for regeneration in an acute murine crush injury model [14]. In translational approaches, incorporation of M2-derived MGEVs into electroconductive hydrogels enhances neural stem cell proliferation, axonal regeneration, and functional recovery in an acute rat transection model of traumatic SCI, highlighting their therapeutic potential [15].

Beyond SCI, MGEVs have been shown to exert both detrimental and protective effects in other CNS conditions [Supplementary references 61–76]. In stroke models, M2 microglia-derived EVs promote white matter repair and angiogenesis via miRNA-mediated pathways, while in neurodegenerative diseases, MGEVs can either propagate pathology through the transfer of toxic proteins and pro-inflammatory signals or exert neuroprotective effects depending on their cargo. Like ADEVs, MGEVs carry proteins, lipids, and nucleic acids that reflect the activation state of their parent microglia. For example, EVs released from pro-inflammatory microglia contain cytokines such as IL-1β and TNF-α and can propagate inflammatory signaling, whereas EVs derived from homeostatic or anti-inflammatory microglia carry regulatory molecules like TGF-β. Similarly, EVs can transport specific microRNAs which modulate gene expression in recipient cells. A study by Santiago et al. explored the molecular profiles of MGEVs under different activation states. EVs derived from LPS-activated MG were shown to induce pro-inflammatory transcriptomic changes in resting responder MG. Similarly, EVs from inflammatory MG carry miR-146a-5p which has been shown to downregulate presynaptic synaptotagmin1 (Syt1) and postsynaptic neuroligin1 (Nlg1) leading to decreased dendritic spine density and synaptic stability of rat primary neurons. In contrast, EVs from non-activated MGs carry miR-140-5p, which has been shown to significantly reduce MG activation and the secretion of pro-inflammatory cytokines [Supplementary references 61–76]. However, these findings are derived from non-SCI models and should be interpreted cautiously when extrapolated to spinal cord injury. An overview of the cargo of MGEVs and their functions is given in Fig. 2c, d.

In summary, the therapeutic potential of MGEVs appears to be heavily influenced by the microglial activation state. Beneficial outcomes are typically associated with EVs derived from MG in a neuroprotective or anti-inflammatory state, while those from pro-inflammatory microglia often exacerbate injury and inflammation. Therefore, the key to effectively utilizing MGEVs as therapeutic tools lies in first engineering or modulating the MG state to ensure the production of EVs with reparative and anti-inflammatory properties prior to their harvest and application as well as finding the appropriate time window after injury for application: In the acute phase, MGEVs primarily propagate neuroinflammation and contribute to secondary injury through pro-inflammatory signaling and increased neuronal apoptosis. In contrast, during the subacute phase, MGEVs from reparative microglial states support recovery by promoting oligodendrogenesis, reducing oxidative stress, and enhancing neuronal survival. These effects are closely linked to their molecular cargo, including microRNAs regulating inflammatory and apoptotic pathways. Accordingly, therapeutic strategies will require temporal control, aiming to limit detrimental EV signaling early after injury while enhancing reparative EV-mediated communication in later phases.

### Oligodendrocytes-derived EVs

Oligodendrocytes (OCs) are the main insulator in the CNS [16]. They extend a handful of processes that wrap around the axons and cover it in myelin leaving Nodes of Ranvier in-between from which the electrical signals jump quickly from the dendrites down the axons to the synapses in saltatory conduction. In contrast to SCs, a single oligodendrocyte can myelinate multiple axon segments, either on one axon or on multiple axons from various neurons. Following SCI, oligodendrocytes are highly vulnerable to secondary injury processes, including excitotoxicity, oxidative stress, and inflammation, leading to demyelination and impaired axonal conduction. OCs and their precursor cells, OPCs, are essential for remyelination after SCI [17]. Remyelination involves OPC activation, proliferation, migration to injury sites, and differentiation into mature OCs that restore myelin sheaths. In addition to their role in remyelination, OPCs contribute to injury responses by modulating inflammation, interacting with other glial cells, and influencing scar formation and tissue remodeling [17].

In contrast to AD- and MGEVs, direct evidence for oligodendrocyte-derived extracellular vesicles (ODEVs) or OPC-derived EVs in traumatic SCI remains limited. Current understanding is therefore largely based on oligodendrocyte-neuron communication studies and indirect evidence from demyelinating disease models. ODEVs are well established mediators of axon-glia communication and support neuronal function by transferring proteins, lipids, and metabolic enzymes (reviewed in [18], Supplementary references 77–91). ODEVs have been shown to enhance neuronal stress resistance and metabolic activity, enabling neurons to better withstand conditions such as oxidative stress or nutrient deprivation. In addition, ODEV cargo can include factors such as SIRT2 and CerS2, which modulate neuronal metabolism and promote neuronal survival under stress conditions. These findings support the concept that ODEVs contribute to axonal maintenance and resilience, processes that are highly relevant in the context of SCI. However, ODEVs are not uniformly beneficial and may also contribute to inhibitory signaling in the injured CNS. Oligodendrocyte-derived EVs contain myelin-associated proteins such as myelin basic protein (MBP), proteolipid protein (PLP), and enzymes such as CNP, reflecting their origin. Importantly, EVs can also carry myelin-associated inhibitory molecules including myelin-associated glycoprotein (MAG), oligodendrocyte myelin glycoprotein and Nogo-A which are known to impede axonal regeneration following CNS injury. These molecules activate signaling pathways, such as the Ras homolog A (RhoA) pathway, which leads to the collapse of the growth cone and inhibits axonal regrowth. The presence of such inhibitory molecules within EVs suggests that oligodendrocyte-derived vesicles may contribute to the non-permissive environment after SCI. Consistent with this, ODEVs have been shown to exert context-dependent effects on oligodendrocyte biology itself. For example, EV-like vesicles released by oligodendrocytes can inhibit oligodendrocyte differentiation and myelin membrane formation through mechanisms involving actomyosin contractility. In addition, in pathological conditions such as multiple sclerosis (MS), ODEVs have been shown to carry pro-inflammatory components, including P2X7R, which is associated with inflammasome activation and cytokine release. These findings further highlight that ODEV function depends on the physiological or pathological state of the parent cell. An overview of the cargo of MGEVs and their functions is given in Fig. 2f.

In the context of SCI, EVs from OPC might be beneficial over ODEVs. Notably, treatments involving OPC-derived EVs in MS models resulted in immune modulation, shifting the lymphocyte profile from Th1 to Th2, something that would be also beneficial after SCI. Additionally, there was a reduction in reactive microgliosis and astrogliosis in the groups treated with EVs, although no reduction in demyelination was observed. Unfortunately, little is known about the contents of OPC-EVs. Future studies are required to define their functional roles in traumatic SCI and to determine whether their beneficial properties can be harnessed for regenerative therapies. Regarding their appropriate application window, oligodendrocyte- and OPC-derived EVs are primarily relevant during the subacute to chronic phases of SCI, where remyelination and axonal regeneration become critical. ODEVs may support repair by modulating inflammation and promoting oligodendrocyte differentiation, thereby enhancing remyelination of demyelinated axons. In contrast, oligodendrocyte-derived EVs can also carry myelin-associated inhibitory proteins, such as Nogo-A, which contribute to growth cone collapse and limit axonal regeneration. This dual role highlights that EV-mediated signaling from the oligodendrocyte lineage can either support or restrict recovery, depending on EV origin and cargo composition. Therefore, therapeutic strategies will likely require selective targeting of OPC-derived EVs or modulation of oligodendrocyte EV cargo to promote remyelination while minimizing inhibitory signaling.

### Schwann-cell derived EVs

Schwann cells (SC) are a key component of the peripheral nervous system as they support axonal growth, myelination, and neural repair. Following nerve injury, SCs undergo transformation into a repair phenotype characterized by the downregulation of myelin-associated genes and the upregulation of trophic factors, cytokines, and debris-clearing mechanisms [19]. This dedifferentiation is mediated by transcription factors including c-Jun and enables SCs to promote axonal regeneration, guide regrowing axons, and facilitate remyelination [19]. Given their significant contribution to nerve regeneration in the peripheral nervous system, SC transplantation has been explored as a therapeutic strategy for SCI. Previous literature has demonstrated that transplanted SCs can enhance axonal regeneration, promote remyelination, modulate inflammatory responses, and improve functional recovery after SCI [20]. Furthermore, SC transplantation aided in attenuating a pro-inflammatory response by reducing the number of pro-inflammatory microglia and macrophages, which adds to their neuroprotective effects.

To explore the therapeutic efficacy of SCEVs, they were first investigated in the peripheral nervous system [see Supplementary references 92–95]. They promote axonal regeneration by modulating cytoskeletal dynamics, including the inhibition of RhoA signaling, thereby reducing axonal retraction and enhancing neurite outgrowth. Furthermore, SCEVs have been shown to support myelination, improve functional recovery, and enhance regenerative responses, particularly when Schwann cells are mechanically stimulated prior to EV isolation. Beyond direct neuronal effects, SCEVs also contribute to the modulation of inflammation and vascular responses, suggesting a broader role in shaping the regenerative microenvironment.

**Fig. 3:**
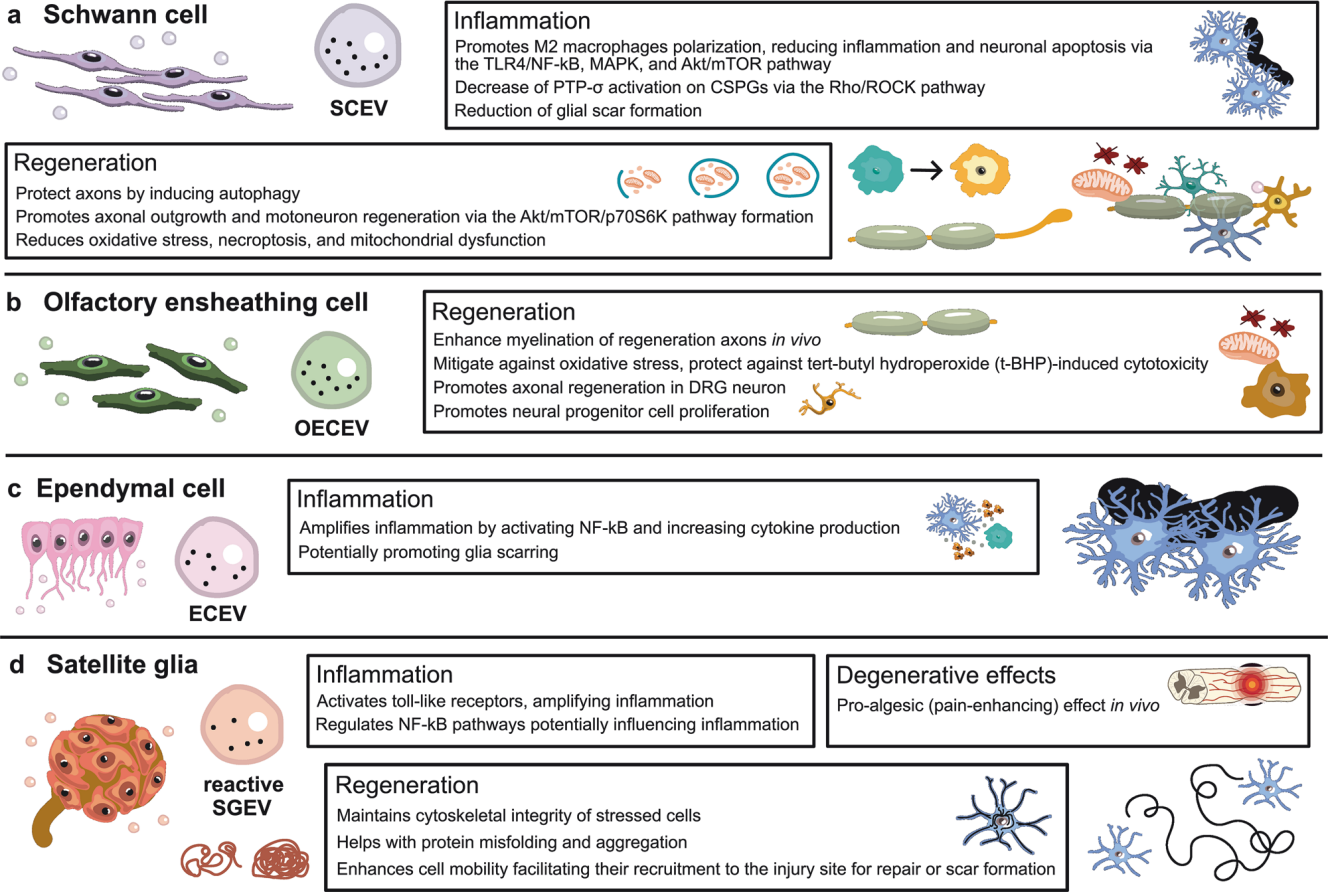
Schwann cell, olfactory ensheathing cells, ependymal and satellite glia-derived EVs and their effects. **a** Effects of EVs derived from Schwann cells. **b** Effects of EVs derived from olfactory ensheathing cells. **c** Cargo and effects of EVs derived from ependymal. **d** Cargo and effects of EVs derived from reactive and not reactive satellite glia cells.

In the context of SCI, emerging evidence indicates that SCEVs exert multifaceted therapeutic effects targeting key pathological processes. Notably, recent work has highlighted a previously underappreciated role of SCEVs in promoting angiogenesis, a critical component of spinal cord repair. Huang et al. [21] demonstrated that SCEVs are internalized by endothelial cells and enhance their proliferation, migration, and tube formation capacity in a mouse contusion model of traumatic SCI. Mechanistically, these effects were mediated by the transfer of integrin-β1 via exosomal cargo, which was required for the pro-angiogenic response and contributed to increased vascular density and improved functional recovery following SCI. This finding is particularly relevant given that vascular disruption and impaired perfusion are central drivers of secondary injury after SCI when administered 30 min post injury. In addition to vascular modulation, SCEVs have been shown to regulate glial and extracellular matrix responses. For instance, SCEVs, injected three times a week over four weeks, increased toll-like receptor 2 on astrocytes leading to reduced deposition of chondroitin sulfate proteoglycans and reduction in glial scar formation in a rat contusion SCI model [22]. Similarly, SCEVs, systemically administered up to 4 weeks post injury, were reported to induce axon growth by decreasing PTP-σ activation on chondroitin sulfate proteoglycans via the Rho/ROCK pathway, which promoted a reduction in scar tissue formation and promoted motor function recovery in a rat contusion model of SCI [23]. At the intracellular level, SCEVs influence multiple survival and repair pathways. SCEVs activate the Akt/mTOR/p70S6K pathway, enhancing neuroplasticity and functional recovery [24] as well as AMPK-mediated mitophagy, which reduces oxidative stress, mitochondrial dysfunction, and necroptosis [25]. A study applying a SCEVs and methylprednisolone-loaded nanofiber patch in an acute rat contusion SCI model demonstrated increased M2-macrophase polarization and decreased neuronal apoptosis [26]. Lastly, SCEVs were also described to promote recovery by inducing autophagy in an acute rat contusion SCI model, which protects axons and enhances motor function recovery through the EGFR/Akt/mTOR signaling pathway [27]. An overview of the cargo of SCEVs and their functions is given in Fig. 3a.

SCEVs show great promise as a therapeutic tool for SCI, offering benefits such as enhanced stability, safety, and bioavailability compared to cell transplantation. They are most relevant during the subacute phase of SCI, where they promote axonal regeneration, modulate the inhibitory extracellular matrix, and support vascular remodeling. By reducing CSPG deposition, enhancing autophagy and mitophagy, and shifting macrophage/microglial polarization toward a reparative phenotype, SCEVs contribute to creating a pro-regenerative microenvironment. In addition, their effects on angiogenesis and neuronal survival suggest a supportive role extending into the early chronic phase. Accordingly, SCEVs represent a promising strategy to enhance regeneration after SCI, particularly when applied after the initial inflammatory phase to support tissue repair and functional recovery.

### Olfactory ensheathing cell-derived EVs

Olfactory ensheathing cells (OECs) are specialized glia found in the olfactory nerve, olfactory mucosa (OM) and the glomerular layers of the olfactory bulb (OB).[28] They support the continuous regeneration of the olfactory neurons by guiding the growing axons, clearing debris and scar tissue and providing partial myelination. Additionally, they secrete neurotrophic factors, including NGF, BDNF, GDNF, and CNTF, and cytokines, most prominently TGFb-2, to limit inflammation, mediate debris removal and enhance neuronal growth and survival [28]. Due to their capacity to modulate inflammation, promote axonal regrowth, and reduce scar tissue, OECs are widely researched in the context of nerve regeneration, particularly within the CNS [29]. The use of autologous OEC transplantation to treat SCIs has shown their capacity to aid neuroregeneration in animal models in both acute and delayed/chronic lesions. In small-scale human trials, both OB- and OM-OEC autotransplantation have been reported to result in partial functional improvements in individuals with clinically complete SCI, classified according to the ASIA/ISCoS ISNCSCI/AIS framework [29].

Direct evidence in SCI is primarily provided by Fan et al. [30], who demonstrated that OECEVs exert immunomodulatory effects in a rodent model of traumatic SCI. In this study, OECEVs were engulfed by microglia and macrophages, leading to suppression of pro-inflammatory signaling pathways, including NF-κB and c-Jun, and promoting a shift toward an anti-inflammatory phenotype. This resulted in reduced neuroinflammation and improved functional recovery after SCI.

The majority of the current OECEVs knowledge is based on supportive evidence from peripheral nerve and in vitro models [Supplementary references 96, 97]. In vitro, OECEVs stimulated dose-dependent axonal growth in dorsal root ganglion cells, with efficacy relying on the structural integrity of the EVs. In vivo, OECEVs incorporated into nerve conduits significantly axonal regeneration, myelination of regenerated axons, and functional neurological recovery in rat models of peripheral nerve injury. Studies with human mucosal OECEVs demonstrate their therapeutic potential by enhancing the proliferation of neural progenitor cells (NPCs) and reduced oxidative stress-induced cytotoxicity, suggesting a potential role in mitigating secondary injury processes such as oxidative damage. An overview of the cargo of OECEVs and their functions is given in Fig. 3b.

Taken together, OECEVs appear to influence key processes relevant to SCI repair, including axonal growth, neuronal survival, and immune modulation. They are likely most effective during the subacute phase of SCI, where modulation of inflammation, promotion of axonal growth, and support of neuronal survival are critical for recovery. By shifting macrophage/microglial polarization toward anti-inflammatory phenotypes and enhancing neurite outgrowth, OECEVs may help limit secondary injury while facilitating regeneration. Their ability to reduce oxidative stress and support neural progenitor cell viability further suggests a role in stabilizing the injury microenvironment. However, given the limited number of SCI-specific studies, these effects should be interpreted cautiously, and further work is required to define their optimal timing and therapeutic application in SCI.

### Ependymal cell-derived EVs

Ependymal cells are specialized glial cells lining the central canal of the spinal cord and the ventricles in the brain. They contribute to cerebrospinal fluid (CSF) homeostasis and form a barrier that supports CNS stability and protection [31]. In response to SCI, ependymal cells exhibit reactive changes, including proliferation and multipotency, suggesting a latent role as stem/progenitor-like capacity [31]. Spinal cord ependymal cells have been shown to contribute to injury responses through glial and, to a limited extent, neuronal lineage differentiation [32]. Interestingly, ependymal cells retain expression of developmental transcription factors like MSX1, ARX, and FOXA2, even in adulthood, indicating preserved plasticity across the lifespan [33].

In contrast to their well-characterized cellular response to SCI, evidence for extracellular vesicles derived from spinal ependymal cells (ECEVs) is extremely limited. Current insights are largely indirect and derived from studies on CSF-derived or choroid plexus-derived EVs rather than spinal ependymal cells themselves (see Supplementary references 98–100). An overview of the cargo of ECEVs and their functions is given in Fig. 3c. EVs from the choroid plexus epithelium contain pro-inflammatory miRNAs, such as miR-146a and miR-155, which are released into the CSF to communicate with CNS-resident cells, including astrocytes and microglia. While these findings suggest that EV-mediated signaling within CSF compartments may influence neuroinflammatory responses, their direct relevance to spinal ependymal cells in SCI remains unclear. Importantly, species-specific differences further complicate translation, as the human spinal cord ependymal region undergoes age-related structural changes, with reduced proliferative capacity and partial replacement by astrocytic and perivascular cell populations. Consequently, it remains uncertain whether ependymal cell-derived EVs play a meaningful role in spinal cord repair in humans. Further research is needed to determine whether ECEVs contribute directly to spinal cord repair or primarily reflect broader CSF-mediated signaling processes following injury.

From what little is known, ECEVs may be most relevant during the acute to subacute phases of SCI, where they could influence inflammatory signaling within the CSF and modulate the early injury microenvironment. Given the role of ependymal cells in injury-induced proliferation and potential lineage plasticity, ECEVs may contribute to intercellular communication that shapes repair processes, including gliosis and tissue remodeling.

### Satellite cell-derived EVs

Satellite glial cells (SGCs) are the predominant glial cells in sensory ganglia, where they closely envelop neuronal somata and regulate the local microenvironment [34]. Beyond their structural role, SGCs exhibit immune-like properties, including including cytokine release, phagocytic activity, and antigen presentation. Following nerve injury, SGCs undergo significant morphological and functional changes, such as upregulation of glial fibrillary acidic protein (GFAP), stronger gap junction coupling, and production of pro-inflammatory mediators. These changes contribute to heightened neuronal excitability and are strongly associated with

Evidence for a role of SGCs in SCI is largely indirect and mediated through their influence on sensory neuron-spinal cord signaling and neuroinflammatory pathways. Activation of SGCs in dorsal root ganglia has been shown to promote pro-inflammatory signaling cascades that can extend to the spinal cord and contribute to microglial activation and persistent pain states. neuropathic pain and central sensitization [35, 36].

EVs derived from SGCs (SGCEVs) have been characterized under both physiological and inflammatory conditions [Supplementary references 101–108], but their role in SCI repair remains poorly defined. Proteomic analyses indicate that inflammatory activation alters SGCEV cargo composition specifically histone H2B, ubiquitin-60S ribosomal, myosin-9, and elongation factor 1-alpha expression which regulate inflammation, cytoskeleton activity, protein folding and stress responses. An overview of the known cargo and effect of SGCEVs and their functions is given in Fig. 3d.

SGCEVs are likely most relevant during the acute phase of SCI, where peripheral and central inflammatory signaling contributes to the development of neuropathic pain and secondary injury. By modulating neuronal excitability and cytokine signaling, SGCEVs may influence early neuroinflammatory responses and pain sensitization. However, current evidence suggests that SGCEVs primarily contribute to pro-nociceptive and pro-inflammatory processes, and their role in regeneration after SCI remains poorly defined. Therefore, therapeutic strategies targeting SGCEVs may focus on limiting their detrimental effects rather than directly promoting repair.

## Effects and targets of glia-cell derived EVs in spinal cord injury

**Fig. 4:**
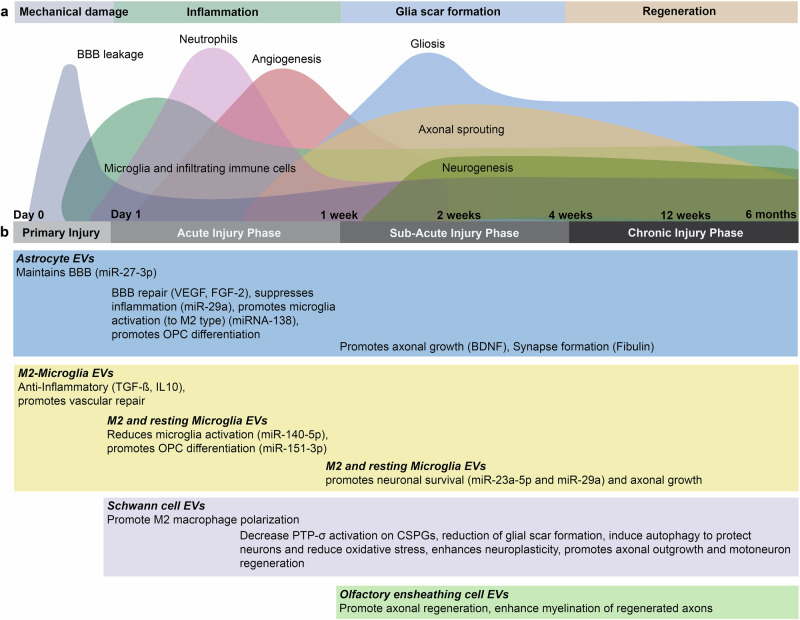
Various stages of SCI. **a** Time-dependent processes after spinal cord injury. **b** Potential timepoints and reasons for when glia cell-derived EVs would be the most effective after spinal cord injury.

As outlined in the previous sections, glial EVs influence SCI pathology through multiple, context-dependent mechanisms that are shaped by their cellular origin, activation state, and the timing of their release. Rather than acting through isolated pathways, these vesicles modulate interconnected processes that evolve dynamically across the different phases of injury. Across studies, the functional impact of glial EVs can be broadly grouped into four overarching, phase-dependent strategies (visualized in Fig. 4 and, together with the effects and cargo of the EVs, summarized in Table 1).

1. **Early vascular stabilization and barrier protection (acute phase):** Immediately after injury, EVs contribute to maintaining blood-spinal cord barrier integrity and restoring vascular function through the actions of ADEVs and M2-MGEVs. This includes endothelial stabilization, reduction of vascular leakage, and early angiogenic signaling, which together help limit secondary damage.
2. **Modulation of neuroinflammation (acute to subacute phase):** A central function of glial EVs is the regulation of immune responses. Depending on their origin and activation state, ADEVs and M2-MGEVs can either amplify pro-inflammatory signaling or promote a shift toward anti-inflammatory and reparative phenotypes as well as promote OPC differentiation, further aiding remyelination processes. SCEVs play a key role in promoting M2 macrophage polarization, thereby reducing inflammation and apoptosis.
3. **Regulation of axonal regeneration and scar-associated inhibition (subacute phase):** As axonal sprouting and scar formation become prominent in the sub-acute injury phase, glia-derived EVs take on neuroprotective roles. SCEVs combat glial scar formation and reduce oxidative stress and mitochondrial dysfunction, promoting motor neuron regeneration.
4. **Support of remyelination and neuroplasticity (subacute to chronic phase):** In later stages, EVs contribute to long-term recovery processes, including oligodendrocyte differentiation, remyelination, synaptic remodeling, and circuit plasticity. OECEVs contribute by enhancing axonal regeneration and the myelination of regenerated axons. Meanwhile, ADEVs support synapse formation and axonal growth. M2-MGEVs play a critical role by promoting neuroprotection and axonal growth, while clearing toxic aggregates.

**Table 1:** Summary of effects of glia-derived EVs in *SCI*.

Key Beneficial Effects	Potential Detrimental Effects	Key Cargo Molecules	SCI Phase of Max Benefit	Evidence Strength in SCI
Astrocytes (ADEVs)				
Maintain BBB integrity (miR-27a-3p), promote OPC differentiation, angiogenesis (VEGF, FGF-2), anti-inflammatory (miR-29a)	Reactive ADEVs may impair neurite outgrowth, spread toxic proteins (Aß, Tau)	Heat shock proteins, ApoE, VEGF, FGF-2, BDNF, miR-27a-3p, miR-29a, miR-138, fibulin-2, miR-92b-3b, CCL7, miR-5121	Acute (BBB protection), Subacute (reduce inflammation, promote angiogenesis), Chronic (synaptic remodeling)	Moderate-strong (two SCI studies, extrapolation from TBI/stroke)
Microglia (MGEVs)				
M2-MGEVs promote remyelination (miR-151-3p), reduce inflammation (miR-140-5p), angiogenesis (TGF-ß, IL-10)	M1-MGEVs amplify inflammation, promote neuronal degeneration, propagate toxic aggregates	TMEM119, neurotrophic factors, P2RY12, miR-29a, TGF-ß, IL-10, miR-151-3p, miR-140-5p, miR-155, miR-23a-5p, pro-inflammatory cytokines (IL-1b, TNF-a), miR-146a-5p, lipids and enzymes	Acute/Subacute (reduce inflammation, promote remyelination), Chronic (clear toxic aggregates)	Strong preclinical SCI evidence for M2-EVs; clear mechanistic pathways identified
Oligodendrocytes (ODEVs)				
Metabolic support to axons (SIRT2), stress resistance, immunomodulation (reduce microgliosis/astrogliosis)	Contain inhibitory myelin proteins (Nogo-A, MAG, OMgp), may impair axon growth	SIRT2, P2X7R, MAG, OMgp, Nogo-A, CerS2	Subacute/Chronic (remyelination, metabolic support)	Weak-moderate (mostly MS/demyelination studies; limited SCI-specific work)
Schwann Cells (SCEVs)				
Reduce glial scar (Rho/ROCK inhibition), promote axon regrowth (Akt/mTOR), enhance neuroplasticity, M2 polarization	Limited evidence of detrimental effects; potential off-target immune activation if systemically delivered	None clearly identified	Subacute (scar reduction), Chronic (axon regeneration, neuroplasticity)	Moderate-strong (multiple SCI animal studies)
Olfactory Ensheathing Cells (OECEVs)				
Promote axon regeneration, enhance myelination, reduce oxidative stress, shift microglia/macrophages to anti-inflammatory state	None clearly identified; limited data on pathological roles	None clearly identified	Subacute/Chronic (axon regeneration, remyelination, anti-inflammatory)	Moderate (mostly peripheral nerve injury studies; some SCI data)
Ependymal Cells (ECEVs)				
Potential modulation of inflammation via miR-146a, miR-155; theoretical regenerative role via stem-like properties	May carry pro-inflammatory cargo in disease states	miR-146a, miR-155	Potentially Subacute (inflammation modulation), uncertain in Chronic phase	Weak (mostly theoretical; little direct SCI evidence)
Satellite Glia Cells (SGCEVs)				
May modulate inflammation, maintain cytoskeleton integrity, possible role in neuroprotection (speculative)	EVs from reactive SGCs may promote pain hypersensitivity, inflammation	Histone H2B, myosin-9, elongation factor 1-alpha, ubiquitin-60S ribosomal	Unclear; potentially Subacute (inflammation modulation)	Weak (speculative; few EV-specific SCI studies)

The table compares the major glial EV subtypes (astrocyte, microglia, OC, SC, OECs, ependymal cell, and SGC), outlining their key beneficial and detrimental effects, representative cargo molecules, phases of SCI where their actions are most relevant, and the relative strength of available evidence.

Importantly, these effects are not uniformly beneficial. The same EV populations can exert opposing functions depending on their cellular state and the timing of their release, underscoring the need for precise temporal and cell-specific targeting.

## Future therapeutic potential of EVs in spinal cord injury

While the potential of glia-derived EVs is undeniable, several challenges need to be overcome to realize their therapeutic and diagnostic promise fully. The lack of standardized methods for EV isolation, characterization, and quantification hinders reproducibility and comparability across studies [37]. Equally important are the models used to test these therapies, as both in vitro and in vivo systems determine how well preclinical findings can be translated to the clinic. Preclinical models are essential to evaluate the safety and efficacy of glia-derived EVs before clinical translation. In vitro systems provide controlled environments to dissect mechanisms such as EV-mediated modulation of inflammation, oxidative stress, or blood–brain barrier (BBB) permeability [38]. Advances in patient-derived induced pluripotent stem cells (iPSCs) have further improved translational relevance, as they can be differentiated into neurons and glial cells to model SCI processes. These platforms not only enable mechanistic studies but may themselves serve as therapeutic tools, since transplantation of iPSCs or their EVs has been shown to support repair. More complex set-ups, including microfluidic devices, 3D hydrogels, organoids, and organotypic spinal cord slices better recapitulate cytoarchitecture and allow investigation of EV effects in acute and sub-acute phases of SCI. However, while indispensable for mechanistic insights and high-throughput testing, in vitro models cannot replicate systemic immune responses or behavioral outcomes, limiting their predictive value for clinical efficacy [Supplementary references 109–121].

Animal models remain critical for preclinical evaluation [39]. Rodents, especially rats, are widely used due to their reproducible pathology and established behavioral assays, while mouse transgenic strains allow for pathway-specific mechanistic studies. Zebrafish can be useful for early EV screening but lack translational fidelity. Larger animals such as pigs and non-human primates offer closer immunological and neuroanatomical parallels to human SCI, but their use is restricted by cost, ethical, and regulatory barriers. Importantly, injury models differ in translational relevance: contusion and compression paradigms best reflect clinical SCI, whereas transection models allow investigation of regeneration and biomaterial strategies but only partly mimic human pathology. Despite inherent limitations, combining advanced human in vitro systems with carefully chosen animal models will remain essential to bridge the gap toward clinical application of EVs [Supplementary references 122–132].

Besides their therapeutic application after SCI, another promising avenue for glia-derived EVs is their potential use as biomarkers [40]. Given their ability to cross the BBB, ADEVs along with neuron- and oligodendrocyte-derived EVs, can be detected in blood samples, offering a minimally invasive approach for diagnosing conditions. For example, elevated GFAP levels in ADEVs at early time points post-stroke (24 h and 7 days) were positively correlated with neurological impairment, suggesting that ADEV GFAP levels could dynamically reflect the severity of neurovascular damage and recovery. Similarly, MGEVs hold immense promise as diagnostic tools for CNS diseases. The presence of specific microRNAs or pathological aggregates in EVs isolated from CSF or blood could provide valuable insights into the disease state and response to therapy. These biomarker capabilities underscore the diagnostic potential of EVs for SCI, particularly in tracking injury progression and recovery which in turn would allow for targeted and customized treatment [Supplementary references 133–134].

Lastly, delivering EV-based therapies to the CNS remains a significant hurdle due to the protective nature of the BBB. Although EVs have been reported to cross the BBB naturally, achieving sufficient concentrations in target regions without off-target effects is a persistent challenge. Safety concerns also arise from the potential off-target effects of engineered EVs and their unintended consequences outside of the CNS. To reduce off-target effects and enhance homing of therapeutic EVs, local application strategies remain the most promising. Here, hydrogels that are investigated to minimize glial scar formation and enable cell migration are being loaded with EVs to support regeneration. This underscores the current understanding that EV therapeutics, while achieving promising preliminary results, are best used in combination with other treatment strategies to achieve its full potential.

Importantly, the past five years have seen rapid progress in the EV field. Advances in high-resolution omics and single-vesicle characterization have enabled the identification of glia-specific EV signatures and cargo relevant to SCI. Preclinical studies now consistently report functional improvements in locomotion, axonal regeneration, and remyelination following administration of astrocyte-, microglia-, and SC-derived EVs in rodent SCI models. Engineered EVs with tailored cargo or enhanced targeting abilities are moving from theoretical concepts to experimental reality, while scalable bioreactor systems for MSC- and neural-derived EVs bring clinical translation closer. Parallel advances in human iPSC-derived models and spinal organoids allow EV testing in human-relevant platforms, bridging the translational gap. Together, these developments demonstrate that EVs are evolving from proof-of-concept interventions into increasingly defined, reproducible therapeutic candidates [Supplementary references 135–137].

## Conclusion

Glial cell-derived EVs present an exciting avenue for SCI treatment, offering a cell-free therapeutic strategy that modulates immune responses, enhances neuroprotection, and supports regeneration. While earlier studies were largely descriptive, recent years have brought a transition toward mechanistic insight, refined engineering strategies, and consistent preclinical efficacy.

In conclusion, the field has progressed from exploring the possibility of EV therapy to defining the parameters for translation. Glia-derived EVs hold promise not only as regenerative tools but also as minimally invasive biomarkers for SCI. With coordinated efforts in standardization, delivery optimization, and integration of preclinical and humanized models, EV-based therapies are poised to become an integral part of next-generation strategies for spinal cord repair.

## Supplementary information

Supplementary references

Supplementary table

## Data Availability

No new datasets were generated or analysed during the current study. All sources discussed in this review are available in the cited literature and supplementary reference list.
