# Supplementary material for: Glia cell-derived extracellular vesicles as modulators in spinal cord injury repair
Source: Spinal Cord. 2026 Jun 9;64(7):588–99. doi: 10.1038/s41393-026-01231-z (PMC13345972; doi:10.1038/s41393-026-01231-z)
Supplement: Supplementary file 1 — Supplementary references [file 41393_2026_1231_MOESM1_ESM.docx]

**Supplementary references**

1. Kretzer RM. A Clinical Perspective and Definition of Spinal Cord Injury. Spine (Phila Pa 1976). 2016;41 Suppl 7:S27.

2. Aarabi B, Albrecht JS, Simard JM, Chryssikos T, Schwartzbauer G, Sansur CA, et al. Trends in Demographics and Markers of Injury Severity in Traumatic Cervical Spinal Cord Injury. J Neurotrauma. 2021;38(6):756-64.

3. Dumont RJ, Okonkwo DO, Verma S, Hurlbert RJ, Boulos PT, Ellegala DB, et al. Acute spinal cord injury, part I: pathophysiologic mechanisms. Clin Neuropharmacol. 2001;24(5):254-64.

4. Ahuja CS, Wilson JR, Nori S, Kotter MRN, Druschel C, Curt A, et al. Traumatic spinal cord injury. Nature Reviews Disease Primers. 2017;3(1):17018.

5. Futch BG, Kouam RW, Ugiliweneza B, Harrop J, Kurpad S, Foster N, et al. Demographics, Mechanism of Injury, and Outcomes for Acute Upper and Lower Cervical Spinal Cord Injuries: An Analysis of 470 Patients in the Prospective, Multi-Center, North American Clinical Trials Network Registry. J Neurotrauma. 2023;40(17-18):1918-27.

6. Harrop JS, Chi JH, Anderson PA, Arnold PM, Dailey AT, Dhall SS, et al. Congress of Neurological Surgeons Systematic Review and Evidence-Based Guidelines on the Evaluation and Treatment of Patients With Thoracolumbar Spine Trauma: Neurological Assessment. Neurosurgery. 2019;84(1):E32-e5.

7. Mattucci S, Speidel J, Liu J, Tetzlaff W, Oxland TR. Temporal Progression of Acute Spinal Cord Injury Mechanisms in a Rat Model: Contusion, Dislocation, and Distraction. J Neurotrauma. 2021;38(15):2103-21.

8. Phillips AA, Krassioukov AV. Contemporary Cardiovascular Concerns after Spinal Cord Injury: Mechanisms, Maladaptations, and Management. J Neurotrauma. 2015;32(24):1927-42.

9. Roberts TT, Leonard GR, Cepela DJ. Classifications In Brief: American Spinal Injury Association (ASIA) Impairment Scale. Clin Orthop Relat Res. 2017;475(5):1499-504.

10. Lili L, Sunnerhagen KS, Rekand T, Alt Murphy M. Participation and autonomy, independence in activities of daily living and upper extremity functioning in individuals with spinal cord injury. Sci Rep. 2024;14(1):9120.

11. Craig A, Nicholson Perry K, Guest R, Tran Y, Dezarnaulds A, Hales A, et al. Prospective study of the occurrence of psychological disorders and comorbidities after spinal cord injury. Arch Phys Med Rehabil. 2015;96(8):1426-34.

12. Sweis R, Biller J. Systemic Complications of Spinal Cord Injury. Curr Neurol Neurosci Rep. 2017;17(2):8.

13. Futch BG, Kouam RW, Ugiliweneza B, Harrop J, Kurpad S, Foster N, et al. Demographics, Mechanism of Injury, and Outcomes for Acute Upper and Lower Cervical Spinal Cord Injuries: An Analysis of 470 Patients in the Prospective, Multi-Center, North American Clinical Trials Network Registry. Journal of Neurotrauma. 2023;40(17-18):1918-27.

14. Hachem LD, Ahuja CS, Fehlings MG. Assessment and management of acute spinal cord injury: From point of injury to rehabilitation. J Spinal Cord Med. 2017;40(6):665-75.

15. Quinones C, Wilson JP, Jr., Kumbhare D, Guthikonda B, Hoang S. Clinical Assessment and Management of Acute Spinal Cord Injury. J Clin Med. 2024;13(19).

16. Zeller SL, Stein A, Frid I, Carpenter AB, Soldozy S, Rawanduzy C, et al. Critical Care of Spinal Cord Injury. Current Neurology and Neuroscience Reports. 2024;24(9):355-63.

17. Fehlings MG, Cadotte DW, Fehlings LN. A series of systematic reviews on the treatment of acute spinal cord injury: a foundation for best medical practice. J Neurotrauma. 2011;28(8):1329-33.

18. Hellenbrand DJ, Quinn CM, Piper ZJ, Morehouse CN, Fixel JA, Hanna AS. Inflammation after spinal cord injury: a review of the critical timeline of signaling cues and cellular infiltration. Journal of Neuroinflammation. 2021;18(1):284.

19. Fehlings MG, Wilson JR, Tetreault LA, Aarabi B, Anderson P, Arnold PM, et al. A Clinical Practice Guideline for the Management of Patients With Acute Spinal Cord Injury: Recommendations on the Use of Methylprednisolone Sodium Succinate. (2192-5682 (Print)).

20. Liu Z, Yang Y, He L, Pang M, Luo C, Liu B, et al. High-dose methylprednisolone for acute traumatic spinal cord injury: A meta-analysis. Neurology. 2019;93(9):e841-e50.

21. Morrison SA, Lorenz D, Eskay CP, Forrest GF, Basso DM. Longitudinal Recovery and Reduced Costs After 120 Sessions of Locomotor Training for Motor Incomplete Spinal Cord Injury. Arch Phys Med Rehabil. 2018;99(3):555-62.

22. Burchiel KJ, Hsu FP. Pain and spasticity after spinal cord injury: mechanisms and treatment. Spine (Phila Pa 1976). 2001;26(24 Suppl):S146-60.

23. Varghese J, Anderson KD, Widerström-Noga E, Mehan U. A Primary Care Provider's Guide to Pain After Spinal Cord Injury: Screening and Management. Top Spinal Cord Inj Rehabil. 2020;26(3):133-43.

24. Verduzco-Gutierrez M, Raghavan P, Pruente J, Moon D, List CM, Hornyak JE, et al. AAPM&R consensus guidance on spasticity assessment and management. Pm r. 2024;16(8):864-87.

25. Jung Y, Breitbart S, Malvea A, Bhatia A, Ibrahim GM, Gorodetsky C. Epidural Spinal Cord Stimulation for Spasticity: a Systematic Review of the Literature. World Neurosurg. 2024;183:227-38.e5.

26. Lazorthes Y, Sol JC, Sallerin B, Verdié JC. The surgical management of spasticity. Eur J Neurol. 2002;9 Suppl 1:35-41; dicussion 53-61.

27. Field-Fote EC. Therapeutic Interventions to Improve Mobility with Spinal Cord Injury Related Upper Motor Neuron Syndromes. Phys Med Rehabil Clin N Am. 2020;31(3):437-53.

28. Fischer G, Bättig L, Stienen MN, Curt A, Fehlings MG, Hejrati N. Advancements in neuroregenerative and neuroprotective therapies for traumatic spinal cord injury. Front Neurosci. 2024;18:1372920.

29. Deatherage Brooke L, Cookson Brad T. Membrane Vesicle Release in Bacteria, Eukaryotes, and Archaea: a Conserved yet Underappreciated Aspect of Microbial Life. Infection and Immunity. 2012;80(6):1948-57.

30. Welsh JA, Goberdhan DCI, O'Driscoll L, Buzas EI, Blenkiron C, Bussolati B, et al. Minimal information for studies of extracellular vesicles (MISEV2023): From basic to advanced approaches. Journal of Extracellular Vesicles. 2024;13(2):e12404.

31. van Niel G, D'Angelo G, Raposo G. Shedding light on the cell biology of extracellular vesicles. Nature Reviews Molecular Cell Biology. 2018;19(4):213-28.

32. Margolis L, Sadovsky Y. The biology of extracellular vesicles: The known unknowns. PLOS Biology. 2019;17(7):e3000363.

33. Carney RP, Mizenko RR, Bozkurt BT, Lowe N, Henson T, Arizzi A, et al. Harnessing extracellular vesicle heterogeneity for diagnostic and therapeutic applications. Nature Nanotechnology. 2025;20(1):14-25.

34. Bjørge IM, Kim SY, Mano JF, Kalionis B, Chrzanowski W. Extracellular vesicles, exosomes and shedding vesicles in regenerative medicine – a new paradigm for tissue repair. Biomaterials Science. 2018;6(1):60-78.

35. Nazerian Y, Nazerian A, Mohamadi-Jahani F, Sodeifi P, Jafarian M, Javadi SAH. Hydrogel-encapsulated extracellular vesicles for the regeneration of spinal cord injury. Frontiers in Neuroscience. 2023;17.

36. Zhang Y, Wu D, Zhou C, Bai M, Wan Y, Zheng Q, et al. Engineered extracellular vesicles for tissue repair and regeneration. Burns & Trauma. 2024;12:tkae062.

37. Song H, Chen X, Hao Y, Wang J, Xie Q, Wang X. Nanoengineering facilitating the target mission: targeted extracellular vesicles delivery systems design. Journal of Nanobiotechnology. 2022;20(1):431.

38. Ziegler JN, Tian C. Engineered Extracellular Vesicles: Emerging Therapeutic Strategies for Translational Applications. International Journal of Molecular Sciences. 2023;24(20):15206.

39. Wang L, Wang D, Ye Z, Xu J. Engineering Extracellular Vesicles as Delivery Systems in Therapeutic Applications. Advanced Science. 2023;10(17):2300552.

40. Kang M, Jordan V, Blenkiron C, Chamley LW. Biodistribution of extracellular vesicles following administration into animals: A systematic review. Journal of Extracellular Vesicles. 2021;10(8):e12085.

41. Driedonks T, Jiang L, Carlson B, Han Z, Liu G, Queen SE, et al. Pharmacokinetics and biodistribution of extracellular vesicles administered intravenously and intranasally to Macaca nemestrina. Journal of Extracellular Biology. 2022;1(10):e59.

42. Zhao S, Di Y, Fan H, Xu C, Li H, Wang Y, et al. Targeted delivery of extracellular vesicles: the mechanisms, techniques and therapeutic applications. Molecular Biomedicine. 2024;5(1):60.

43. Shanthi KB, Fischer D, Sharma A, et al. Human Adult Astrocyte Extracellular Vesicle Transcriptomics Study Identifies Specific RNAs Which Are Preferentially Secreted as EV Luminal Cargo. Genes (Basel). Mar 31 2023;14(4)doi:10.3390/genes14040853

44. Chun C, Smith AST, Kim H, et al. Astrocyte-derived extracellular vesicles enhance the survival and electrophysiological function of human cortical neurons in vitro. Biomaterials. Apr 2021;271:120700. doi:10.1016/j.biomaterials.2021.120700

45. Jiang S, Li X, Li Y, et al. APOE from patient-derived astrocytic extracellular vesicles alleviates neuromyelitis optica spectrum disorder in a mouse model. Sci Transl Med. Feb 28 2024;16(736):eadg5116. doi:10.1126/scitranslmed.adg5116

46. Upadhya R, Zingg W, Shetty S, Shetty AK. Astrocyte-derived extracellular vesicles: Neuroreparative properties and role in the pathogenesis of neurodegenerative disorders. J Control Release. Jul 10 2020;323:225-239. doi:10.1016/j.jconrel.2020.04.017

47. Wang X, Li A, Fan H, Li Y, Yang N, Tang Y. Astrocyte-Derived Extracellular Vesicles for Ischemic Stroke: Therapeutic Potential and Prospective. Aging Dis. May 7 2024;15(3):1227-1254. doi:10.14336/ad.2023.0823-1

48. Yang ZL, Liang ZY, Lin YK, et al. Efficacy of extracellular vesicles of different cell origins in traumatic brain injury: A systematic review and network meta-analysis. Front Neurosci. 2023;17:1147194. doi:10.3389/fnins.2023.1147194

49. Wu X, Liu H, Hu Q, et al. Astrocyte-Derived Extracellular Vesicular miR-143-3p Dampens Autophagic Degradation of Endothelial Adhesion Molecules and Promotes Neutrophil Transendothelial Migration after Acute Brain Injury. Advanced Science. 2024/02/01 2024;11(5):2305339. doi:<https://doi.org/10.1002/advs.202305339>

50. Hering C, Shetty AK. Extracellular Vesicles Derived From Neural Stem Cells, Astrocytes, and Microglia as Therapeutics for Easing TBI-Induced Brain Dysfunction. Stem Cells Transl Med. Mar 17 2023;12(3):140-153. doi:10.1093/stcltm/szad004

51. Hou Y, Xie Y, Liu X, Chen Y, Zhou F, Yang B. Oxygen glucose deprivation-pretreated astrocyte-derived exosomes attenuates intracerebral hemorrhage (ICH)-induced BBB disruption through miR-27a-3p /ARHGAP25/Wnt/β-catenin axis. Fluids Barriers CNS. Jan 19 2024;21(1):8. doi:10.1186/s12987-024-00510-2

52. Patel MR, Weaver AM. Astrocyte-derived small extracellular vesicles promote synapse formation via fibulin-2-mediated TGF-β signaling. Cell Rep. Mar 9 2021;34(10):108829. doi:10.1016/j.celrep.2021.108829

53. Liao K, Niu F, Hu G, et al. Morphine-mediated release of miR-138 in astrocyte-derived extracellular vesicles promotes microglial activation. J Extracell Vesicles. Oct 2020;10(1):e12027. doi:10.1002/jev2.12027

54. Liu X, Lv X, Liu Z, Zhang M, Leng Y. MircoRNA-29a in Astrocyte-derived Extracellular Vesicles Suppresses Brain Ischemia Reperfusion Injury via TP53INP1 and the NF-κB/NLRP3 Axis. Cell Mol Neurobiol. Jul 2022;42(5):1487-1500. doi:10.1007/s10571-021-01040-3

55. Dutta D, Khan N, Wu J, Jay SM. Extracellular Vesicles as an Emerging Frontier in Spinal Cord Injury Pathobiology and Therapy. Trends Neurosci. Jun 2021;44(6):492-506. doi:10.1016/j.tins.2021.01.003

56. White KE, Bailey HL, Shaw BS, et al. A convenient model of serum-induced reactivity of human astrocytes to investigate astrocyte-derived extracellular vesicles. Original Research. Frontiers in Cellular Neuroscience. 2024-June-10 2024;18doi:10.3389/fncel.2024.1414142

57. You Y, Borgmann K, Edara VV, Stacy S, Ghorpade A, Ikezu T. Activated human astrocyte-derived extracellular vesicles modulate neuronal uptake, differentiation and firing. J Extracell Vesicles. 2020;9(1):1706801. doi:10.1080/20013078.2019.1706801

58. Zhu Y, Wang F, Xia Y, et al. Research progress on astrocyte-derived extracellular vesicles in the pathogenesis and treatment of neurodegenerative diseases. Rev Neurosci. Dec 17 2024;35(8):855-875. doi:10.1515/revneuro-2024-0043

59. Li B, Ma Z, Li Z. A novel regulator in Alzheimer's disease progression: The astrocyte-derived extracellular vesicles. Ageing Research Reviews. 2023/04/01/ 2023;86:101871. doi:<https://doi.org/10.1016/j.arr.2023.101871>

60. Li Z, Moniruzzaman M, Dastgheyb RM, et al. Astrocytes deliver CK1 to neurons via extracellular vesicles in response to inflammation promoting the translation and amyloidogenic processing of APP. J Extracell Vesicles. Dec 2020;10(2):e12035. doi:10.1002/jev2.12035

61. Kannan M, Singh S, Chemparathy DT, et al. HIV-1 Tat induced microglial EVs leads to neuronal synaptodendritic injury: microglia-neuron cross-talk in NeuroHIV. Extracell Vesicles Circ Nucl Acids. 2022;3(2):133-149. doi:10.20517/evcna.2022.14

62. Santiago JV, Natu A, Ramelow CC, et al. Identification of State-Specific Proteomic and Transcriptomic Signatures of Microglia-Derived Extracellular Vesicles. Mol Cell Proteomics. Dec 2023;22(12):100678. doi:10.1016/j.mcpro.2023.100678

63. Grimaldi A, Serpe C, Chece G, et al. Microglia-Derived Microvesicles Affect Microglia Phenotype in Glioma. Front Cell Neurosci. 2019;13:41. doi:10.3389/fncel.2019.00041

64. Lemaire Q, Raffo-Romero A, Arab T, et al. Isolation of microglia-derived extracellular vesicles: towards miRNA signatures and neuroprotection. J Nanobiotechnology. Dec 4 2019;17(1):119. doi:10.1186/s12951-019-0551-6

65. Wang P, Dong S, Liu F, Liu A, Wang Z. MicroRNA-140-5p shuttled by microglia-derived extracellular vesicles attenuates subarachnoid hemorrhage-induced microglia activation and inflammatory response via MMD downregulation. Exp Neurol. Jan 2023;359:114265. doi:10.1016/j.expneurol.2022.114265

66. Raffaele S, Lombardi M, Verderio C, Fumagalli M. TNF Production and Release from Microglia via Extracellular Vesicles: Impact on Brain Functions. Cells. Sep 23 2020;9(10)doi:10.3390/cells9102145

67. Trotta T, Panaro MA, Cianciulli A, Mori G, Di Benedetto A, Porro C. Microglia-derived extracellular vesicles in Alzheimer's Disease: A double-edged sword. Biochem Pharmacol. Feb 2018;148:184-192. doi:10.1016/j.bcp.2017.12.020

68. Ceccarelli L, Giacomelli C, Marchetti L, Martini C. Microglia extracellular vesicles: focus on molecular composition and biological function. Biochem Soc Trans. Aug 27 2021;49(4):1779-1790. doi:10.1042/bst20210202

69. Cohn W, Melnik M, Huang C, et al. Multi-Omics Analysis of Microglial Extracellular Vesicles From Human Alzheimer's Disease Brain Tissue Reveals Disease-Associated Signatures. Front Pharmacol. 2021;12:766082. doi:10.3389/fphar.2021.766082

70. Li Y, Liu Z, Song Y, et al. M2 microglia-derived extracellular vesicles promote white matter repair and functional recovery via miR-23a-5p after cerebral ischemia in mice. Theranostics. 2022;12(7):3553-3573. doi:10.7150/thno.68895

71. Yan B, Liao P, Liu Y, et al. Therapeutic potential of microglia-derived extracellular vesicles in ischemic stroke. Int Immunopharmacol. Sep 30 2024;139:112712. doi:10.1016/j.intimp.2024.112712

72. Wies Mancini VSB, Mattera VS, Pasquini JM, Pasquini LA, Correale JD. Microglia-derived extracellular vesicles in homeostasis and demyelination/remyelination processes. J Neurochem. Jan 2024;168(1):3-25. doi:10.1111/jnc.16011

73. Lombardi M, Parolisi R, Scaroni F, et al. Detrimental and protective action of microglial extracellular vesicles on myelin lesions: astrocyte involvement in remyelination failure. Acta Neuropathol. Dec 2019;138(6):987-1012. doi:10.1007/s00401-019-02049-1

74. Qi Z, Yu Y, Su Y, Cao B, Shao H, Yang JJ. M1-Type Microglia-Derived Extracellular Vesicles Overexpressing IL-1R1 Promote Postoperative Cognitive Dysfunction by Regulating Neuronal Inflammation. Inflammation. Dec 2023;46(6):2254-2269. doi:10.1007/s10753-023-01875-6

75. Zhang Y, Xu C, Nan Y, Nan S. Microglia-Derived Extracellular Vesicles Carrying miR-711 Alleviate Neurodegeneration in a Murine Alzheimer's Disease Model by Binding to Itpkb. Front Cell Dev Biol. 2020;8:566530. doi:10.3389/fcell.2020.566530

76. Li N, Huang Y, Wu Y, Wang Q, Ji P. Extracellular vesicles derived from monomeric α-synuclein-treated microglia ameliorate neuroinflammation by delivery of miRNAs targeting PRAK. Neurosci Lett. Jan 1 2024;818:137562. doi:10.1016/j.neulet.2023.137562

77. Li S, Sheng ZH. Oligodendrocyte-derived transcellular signaling regulates axonal energy metabolism. Curr Opin Neurobiol. Jun 2023;80:102722. doi:10.1016/j.conb.2023.102722

78. Zhang H, Xie XH, Xu SX, et al. Oligodendrocyte-derived exosomes-containing SIRT2 ameliorates depressive-like behaviors and restores hippocampal neurogenesis and synaptic plasticity via the AKT/GSK-3β pathway in depressed mice. CNS Neurosci Ther. Mar 2024;30(3):e14661. doi:10.1111/cns.14661

79. Fröhlich D, Kuo WP, Frühbeis C, et al. Multifaceted effects of oligodendroglial exosomes on neurons: impact on neuronal firing rate, signal transduction and gene regulation. Philos Trans R Soc Lond B Biol Sci. Sep 26 2014;369(1652)doi:10.1098/rstb.2013.0510

80. Frühbeis C, Fröhlich D, Kuo WP, et al. Neurotransmitter-triggered transfer of exosomes mediates oligodendrocyte-neuron communication. PLoS Biol. Jul 2013;11(7):e1001604. doi:10.1371/journal.pbio.1001604

81. Agliardi C, Guerini FR, Zanzottera M, et al. Increased concentrations of P2X7R in oligodendrocyte derived extracellular vesicles of Multiple sclerosis patients. Neurobiol Dis. Sep 2024;199:106601. doi:10.1016/j.nbd.2024.106601

82. Agliardi C, Guerini FR, Zanzottera M, et al. Myelin Basic Protein in Oligodendrocyte-Derived Extracellular Vesicles as a Diagnostic and Prognostic Biomarker in Multiple Sclerosis: A Pilot Study. Int J Mol Sci. Jan 3 2023;24(1)doi:10.3390/ijms24010894

83. Bakhti M, Winter C, Simons M. Inhibition of myelin membrane sheath formation by oligodendrocyte-derived exosome-like vesicles. J Biol Chem. Jan 7 2011;286(1):787-96. doi:10.1074/jbc.M110.190009

84. Casadomé-Perales Á, Naya S, Fernández-Martínez E, et al. Neuronal Prosurvival Role of Ceramide Synthase 2 by Olidogendrocyte-to-Neuron Extracellular Vesicle Transfer. Int J Mol Sci. Mar 22 2023;24(6)doi:10.3390/ijms24065986

85. Casella G, Rasouli J, Boehm A, et al. Oligodendrocyte-derived extracellular vesicles as antigen-specific therapy for autoimmune neuroinflammation in mice. Sci Transl Med. Nov 4 2020;12(568)doi:10.1126/scitranslmed.aba0599

86. Mukherjee N, Ghosh S. Myelin Associated Inhibitory Proteins as a Therapeutic Target for Healing of CNS Injury. ACS Chem Neurosci. Jun 17 2020;11(12):1699-1700. doi:10.1021/acschemneuro.0c00280

87. Sekine Y, Lindborg JA, Strittmatter SM. A proteolytic C-terminal fragment of Nogo-A (reticulon-4A) is released in exosomes and potently inhibits axon regeneration. J Biol Chem. Feb 21 2020;295(8):2175-2183. doi:10.1074/jbc.RA119.009896

88. Rust R, Holm MM, Egger M, et al. Nogo-A is secreted in extracellular vesicles, occurs in blood and can influence vascular permeability. J Cereb Blood Flow Metab. Jun 2024;44(6):938-954. doi:10.1177/0271678x231216270

89. Schwab ME, Strittmatter SM. Nogo limits neural plasticity and recovery from injury. Curr Opin Neurobiol. Aug 2014;27:53-60. doi:10.1016/j.conb.2014.02.011

90. Schmandke A, Schmandke A, Schwab ME. Nogo-A: Multiple Roles in CNS Development, Maintenance, and Disease. Neuroscientist. Aug 2014;20(4):372-386. doi:10.1177/1073858413516800

91. Santos SIP, Ortiz-Peñuela SJ, de Paula Filho A, et al. Oligodendrocyte precursor cell-derived exosomes combined with cell therapy promote clinical recovery by immunomodulation and gliosis attenuation. Front Cell Neurosci. 2024;18:1413843. doi:10.3389/fncel.2024.1413843

92. Lopez-Verrilli MA, Picou F, Court FA. Schwann cell-derived exosomes enhance axonal regeneration in the peripheral nervous system. Glia. Nov 2013;61(11):1795-806. doi:10.1002/glia.22558

93. Yu M, Gu G, Cong M, et al. Repair of peripheral nerve defects by nerve grafts incorporated with extracellular vesicles from skin-derived precursor Schwann cells. Acta Biomater. Oct 15 2021;134:190-203. doi:10.1016/j.actbio.2021.07.026

94. Xia B, Gao J, Li S, et al. Mechanical stimulation of Schwann cells promote peripheral nerve regeneration via extracellular vesicle-mediated transfer of microRNA 23b-3p. Theranostics. 2020;10(20):8974-8995. doi:10.7150/thno.44912

95. Lopez-Leal R, Court FA. Schwann Cell Exosomes Mediate Neuron-Glia Communication and Enhance Axonal Regeneration. Cell Mol Neurobiol. Apr 2016;36(3):429-36. doi:10.1007/s10571-015-0314-3

96. Xia B, Gao J, Li S, et al. Extracellular Vesicles Derived From Olfactory Ensheathing Cells Promote Peripheral Nerve Regeneration in Rats. Original Research. Frontiers in Cellular Neuroscience. 2019-December-06 2019;13doi:10.3389/fncel.2019.00548

97. Tu YK, Hsueh YH. Extracellular vesicles isolated from human olfactory ensheathing cells enhance the viability of neural progenitor cells. Neurol Res. Nov 2020;42(11):959-967. doi:10.1080/01616412.2020.1794371

98. Balusu S, Van Wonterghem E, De Rycke R, et al. Identification of a novel mechanism of blood-brain communication during peripheral inflammation via choroid plexus-derived extracellular vesicles. EMBO Mol Med. Oct 2016;8(10):1162-1183. doi:10.15252/emmm.201606271

99. Garcia-Ovejero D, Arevalo-Martin A, Paniagua-Torija B, et al. The ependymal region of the adult human spinal cord differs from other species and shows ependymoma-like features. Brain. Jun 2015;138(Pt 6):1583-97. doi:10.1093/brain/awv089

100. Paniagua-Torija B, Norenberg M, Arevalo-Martin A, et al. Cells in the adult human spinal cord ependymal region do not proliferate after injury. The Journal of Pathology. 2018/12/01 2018;246(4):415-421. doi:<https://doi.org/10.1002/path.5151>

101. Hanani M, Spray DC. Emerging importance of satellite glia in nervous system function and dysfunction. Nat Rev Neurosci. Sep 2020;21(9):485-498. doi:10.1038/s41583-020-0333-z

102. Pannese E. Biology and Pathology of Perineuronal Satellite Cells in Sensory Ganglia. 2018:1-63.

103. Hanani M, Blum E, Liu S, Peng L, Liang S. Satellite glial cells in dorsal root ganglia are activated in streptozotocin-treated rodents. J Cell Mol Med. Dec 2014;18(12):2367-71. doi:10.1111/jcmm.12406

104. Caruso Bavisotto C, Scalia F, Marino Gammazza A, et al. Extracellular Vesicle-Mediated Cell⁻Cell Communication in the Nervous System: Focus on Neurological Diseases. Int J Mol Sci. Jan 20 2019;20(2)doi:10.3390/ijms20020434

105. Duroux M, Vinterhøj HSH, Stensballe A, Gazerani P. Characterization of released exosomes from satellite glial cells under normal and inflammatory conditions. Scandinavian Journal of Pain. 2017;16(1):170-170. doi:doi:10.1016/j.sjpain.2017.04.018

106. Vinterhøj HSH, Stensballe A, Duroux M, Gazerani P. Characterization of rat primary trigeminal satellite glial cells and associated extracellular vesicles under normal and inflammatory conditions. Journal of Proteomics. 2019/01/06/ 2019;190:27-34. doi:<https://doi.org/10.1016/j.jprot.2018.03.013>

107. Gazerani P. Contribution of Small Extracellular Vesicles from Schwann Cells and Satellite Glial Cells to Pain Processing. Neuroglia. 2024;5(1):1-12. doi:10.3390/neuroglia5010001

108. Zhao L, Liu S, Zhang X, et al. Satellite glial cell-secreted exosomes after in-vitro oxaliplatin treatment presents a pro-nociceptive effect for dorsal root ganglion neurons and induce mechanical hypersensitivity in naïve mice. Molecular and Cellular Neuroscience. 2023/09/01/ 2023;126:103881. doi:<https://doi.org/10.1016/j.mcn.2023.103881>

109. Chen CC, Liu L, Ma F, Wong CW, Guo XE, Chacko JV, et al. Elucidation of Exosome Migration across the Blood-Brain Barrier Model In Vitro. Cell Mol Bioeng. 2016;9(4):509-29.

110. Morad G, Carman CV, Hagedorn EJ, Perlin JR, Zon LI, Mustafaoglu N, et al. Tumor-Derived Extracellular Vesicles Breach the Intact Blood-Brain Barrier via Transcytosis. ACS Nano. 2019;13(12):13853-65.

111. Tomatis F, Rosa S, Simões S, Barão M, Jesus C, Novo J, et al. Engineering extracellular vesicles to transiently permeabilize the blood–brain barrier. Journal of Nanobiotechnology. 2024;22(1):747.

112. Hosoya M, Czysz K. Translational Prospects and Challenges in Human Induced Pluripotent Stem Cell Research in Drug Discovery. Cells. 2016;5(4).

113. Montgomery A, Wong A, Gabers N, Willerth SM. Engineering personalized neural tissue by combining induced pluripotent stem cells with fibrin scaffolds. Biomater Sci. 2015;3(2):401-13.

114. Saglam-Metiner P, Duran E, Sabour-Takanlou L, Biray-Avci C, Yesil-Celiktas O. Differentiation of Neurons, Astrocytes, Oligodendrocytes and Microglia From Human Induced Pluripotent Stem Cells to Form Neural Tissue-On-Chip: A Neuroinflammation Model to Evaluate the Therapeutic Potential of Extracellular Vesicles Derived from Mesenchymal Stem Cells. Stem Cell Reviews and Reports. 2024;20(1):413-36.

115. Shibata T, Tashiro S, Nakamura M, Okano H, Nagoshi N. A Review of Treatment Methods Focusing on Human Induced Pluripotent Stem Cell-Derived Neural Stem/Progenitor Cell Transplantation for Chronic Spinal Cord Injury. Medicina (Kaunas). 2023;59(7).

116. Yu T, Yang L-L, Zhou Y, Wu M-F, Jiao J-H. Exosome-mediated repair of spinal cord injury: a promising therapeutic strategy. Stem Cell Research & Therapy. 2024;15(1):6.

117. Lucia S, Juraj B, Miriam N, Eva S, Dasa C. In Vitro Models of Spinal Cord Injury. In: Heidi F, Monte G, editors. Recovery of Motor Function Following Spinal Cord Injury. Rijeka: IntechOpen; 2016. p. Ch. 2.

118. Shrirao AB, Kung FH, Omelchenko A, Schloss RS, Boustany NN, Zahn JD, et al. Microfluidic platforms for the study of neuronal injury in vitro. Biotechnol Bioeng. 2018;115(4):815-30.

119. Cui B, Liu C, Zhang G, Liu C, Yang F, Hao J, et al. Microfluidics-Based Technologies for Extracellular Vesicle Research. In: Wang Q, Zheng L, editors. Extracellular Vesicles: From Bench to Bedside. Singapore: Springer Nature Singapore; 2024. p. 125-51.

120. Mei Q, Yuen H-Y, Zhao X. Mechanical stretching of 3D hydrogels for neural stem cell differentiation. Bio-Design and Manufacturing. 2022;5(4):714-28.

121. Ju Y, Hu Y, Yang P, Xie X, Fang B. Extracellular vesicle-loaded hydrogels for tissue repair and regeneration. Mater Today Bio. 2023;18:100522.

122. Sharif-Alhoseini M, Khormali M, Rezaei M, Safdarian M, Hajighadery A, Khalatbari MM, et al. Animal models of spinal cord injury: a systematic review. Spinal Cord. 2017;55(8):714-21.

123. Bhalala OG, Pan L, Sahni V, McGuire TL, Gruner K, Tourtellotte WG, et al. microRNA-21 regulates astrocytic response following spinal cord injury. J Neurosci. 2012;32(50):17935-47.

124. Chen G, Li S, Tong K, Huang Z, Liu S, Zhu H, et al. Extracellular vesicles released by transforming growth factor-beta 1-preconditional mesenchymal stem cells promote recovery in mice with spinal cord injury. Bioactive materials. 2024;35:135-49.

125. Sambur DB, Kalinina OV, Aquino AD, Tirikova PV, Koroleva EE, Trulioff AS, et al. Controlling the Immune Response to Zebrafish Spinal Cord Injury via Extracellular Vesicles Secreted by Activated Monocyte-like Cells. Journal of Evolutionary Biochemistry and Physiology. 2024;60(1):84-103.

126. Saraswathy VM, Zhou L, Mokalled MH. Single-cell analysis of innate spinal cord regeneration identifies intersecting modes of neuronal repair. Nature Communications. 2024;15(1):6808.

127. Olaya AMS, Almeida FM, Martinez AMB, Marques SA. Treatment of spinal cord injury with biomaterials and stem cell therapy in non-human primates and humans. Neural Regen Res. 2025;20(2):343-53.

128. Nobunaga AI, Go BK, Karunas RB. Recent demographic and injury trends in people served by the model spinal cord injury care systems. Archives of Physical Medicine and Rehabilitation. 1999;80(11):1372-82.

129. Ashmwe M, Posa K, Rührnößl A, Heinzel JC, Heimel P, Mock M, et al. Effects of Extracorporeal Shockwave Therapy on Functional Recovery and Circulating miR-375 and miR-382-5p after Subacute and Chronic Spinal Cord Contusion Injury in Rats. Biomedicines. 2022;10(7).

130. Park JH, Kim JH, Oh S-K, Baek SR, Min J, Kim YW, et al. Analysis of equivalent parameters of two spinal cord injury devices: the New York University impactor versus the Infinite Horizon impactor. Spine J. 2016;16(11):1392-403.

131. Zeng X, Wei QS, Ye JC, Rao JH, Zheng MG, Ma YH, et al. A biocompatible gelatin sponge scaffold confers robust tissue remodeling after spinal cord injury in a non-human primate model. Biomaterials. 2023;299:122161.

132. Stewart AN, Gensel JC, Jones L, Fouad K. Challenges in Translating Regenerative Therapies for Spinal Cord Injury. Top Spinal Cord Inj Rehabil. 2023;29(Suppl):23-43.

133. Wang P, Lan G, Xu B, Yu Z, Tian C, Lei X, et al. α-Synuclein-carrying astrocytic extracellular vesicles in Parkinson pathogenesis and diagnosis. Transl Neurodegener. 2023;12(1):40.

134. Forró T, Manu DR, Băjenaru OL, Bălașa R. GFAP as Astrocyte-Derived Extracellular Vesicle Cargo in Acute Ischemic Stroke Patients-A Pilot Study. Int J Mol Sci. 2024;25(11).

135. Santiago JV, Natu A, Ramelow CC, Rayaprolu S, Xiao H, Kumar V, et al. Identification of State-Specific Proteomic and Transcriptomic Signatures of Microglia-Derived Extracellular Vesicles. Molecular & Cellular Proteomics. 2023;22(12).

136. Mendt M, Kamerkar S, Sugimoto H, McAndrews KM, Wu CC, Gagea M, et al. Generation and testing of clinical-grade exosomes for pancreatic cancer. JCI Insight. 2018;3(8).

137. Hsiung N, Ju Y, Yang K, Yang P, Zeng W, Zhao H, et al. Organoid-based tissue engineering for advanced tissue repair and reconstruction. Materials Today Bio. 2025;33:102093.
