# Supplementary material for: Glia cell-derived extracellular vesicles as modulators in spinal cord injury repair
Source: Spinal Cord. 2026 Jun 9;64(7):588–99. doi: 10.1038/s41393-026-01231-z (PMC13345972; doi:10.1038/s41393-026-01231-z)
Supplement: Supplementary file 2 — Supplementary table [file 41393_2026_1231_MOESM2_ESM.docx]

**Supplementary table 1: Appraisal of SCI-specific *in vivo* studies.** ADEV: Astrocyte-derived EV, MGEV: Microglia-derived EV, SCEV: Schwann cell-derived EV, OECEV: Olfactory ensheathing cell-derived EV; IV: intravenous, BMS: Basso Mouse Scale, BBB:  [Basso, Beattie, and Bresnahan](https://www.google.com/search?q=Basso%2C+Beattie%2C+and+Bresnahan+%28BBB%29+scale&sca_esv=fd50da422e8f4301&sxsrf=ANbL-n4xh1_TyXHwqA7O5zKk02jbGTEkIg%3A1775980251595&ei=207baYqJJMC_xc8P9uTIiAc&ved=2ahUKEwjJtYSI6ueTAxXVQ_EDHTLgGtUQgK4QegQIARAB&uact=5&oq=BBB+basso+spinal+cord+injury&gs_lp=Egxnd3Mtd2l6LXNlcnAiHEJCQiBiYXNzbyBzcGluYWwgY29yZCBpbmp1cnkyBRAAGO8FMgUQABjvBTIFEAAY7wVI4QpQhgNYyQdwAXgAkAEAmAGQAaABoQaqAQMyLjW4AQPIAQD4AQGYAgOgAuYBwgIKEAAYsAMY1gQYR8ICBhAAGA0YHsICCBAAGAgYDRgewgIIEAAYgAQYogSYAwCIBgGQBgWSBwMxLjKgB_cZsgcDMC4yuAfjAcIHBTAuMi4xyAcJgAgA&sclient=gws-wiz-serp&mstk=AUtExfCOjddBsoLZNfVhrk36qtY2YC3RT6uOMwdA242iuJ30EjeGPPPDkGkL76Ltt7wU1URTl4mPaTewGxhokBEpPdatyRnlQvidJX7KjfUYRIx3byx5wGrWkyuI27pc9rM27JZ9H3GZW1SmKsOVEdSLj0drAUTTg-e1iX5hZVD_YxLfuGF3YuG3v_CE46Y2su9GhkGXNtQ2eYlOG8ILYK8R6mwMCQ&csui=3) scale, TEM: Transmission Electron Microscope, NTA: Nanoparticle Tracking Analysis, WB: Western blot

| **Study** | **Cell** | **Species** | **Injury type** | **Timing & route** | **Randomization** | **Blinding** | **EV characterization** | **Key strength** | **Key limitation** |
| --- | --- | --- | --- | --- | --- | --- | --- | --- | --- |
| **Zhu 2025** | ADEV | Mouse (C57BL/6) | Contusion (T10, NYU) | IV, repeated (2 h → 7 d) | reported | BMS blinded | Very strong (TEM, NTA, western blot; multiple markers, negative markers) | Includes beneficial vs detrimental EVs (CCL7 axis) | No clear power calc; histology blinding unclear |
| **Wang 2025** | ADEV | Mouse (C57BL/6) | Contusion (T10, weight-drop) | IV, single dose | reported | unclear | Very strong (TEM, NTA, multiple markers, purity metric) | Mechanistic miR-5121 / BSCB repair | Timing precision + blinding unclear |
| **Li 2021** | MGEV | Mouse (C57BL/6) | Contusion (T10, NYU) | IV, repeated (3 d) | unclear | BMS blinded | Strong (TEM, NTA, WB, EV markers CD9, CD63, Tsg101) | Strong mechanistic p53/p21/CDK1 pathway | Randomization not clearly stated |
| **Peng 2021** | MGEV | Mouse (C57BL/6) | Contusion (T10, Allen) | IV, repeated | reported | BMS blinded | Strong (TEM, NTA, WB, EV markers CD9, CD63, Tsg101) | Angiogenesis + oxidative stress + electrophysiology | Full blinding of histology unclear |
| **Zhang 2024** | MGEV | Mouse (C57BL/6) | Crush (T8) | IV, repeated | reported | blinded (BMS + footprint) | Strong (TEM, NTA, WB, EV markers CD9, CD63, Tsg101) | Links microglia → astrocyte phenotype (A1 suppression) | Dose schedule needs confirmation |
| **Guan 2024** | MGEV | Rat (SD) | Transection (T8–T10) | Local hydrogel implant | reported | BBB blinded | Strong (TEM, NTA, WB, EV markers CD9, CD63, Tsg101) | Biomaterial + EV synergy (translational angle) | Not directly comparable to contusion studies |
| **Huang 2022** | SCEV | Rat (SD) | Contusion (T10) | IV, early (30 min) | reported | unclear | Moderate-strong (TEM, NTA, WB) | Angiogenesis mechanism (integrin β1) | Blinding + power calc unclear |
| **Pan 2021** | SCEV | Mouse | Crush (T10) | IV, repeated acute → chronic dosing) | reported | blinded | Moderate (TEM, WB) | Clear CSPG/TLR2 mechanism + genetic validation | Model differs from contusion |
| **Zhu 2024** | SCEV | Mouse (C57BL/6) | Compression/crush (T10) | IV (acute → chronic dosing) | reported | blinded (multi-observer) | Moderate (TEM, WB) | Best-defined phase-dependent dosing | Histology blinding unclear |
| **Zhu 2023** | SCEV (+drug) | Rat | Contusion (location not reported) | Local patch (acute) | unclear | unclear | Moderate-strong Moderate (TEM, WB, EV markers CD63, CD9, TSG101) | Translational composite scaffold | Not EV-only → attribution unclear |
| **Xu 2023** | SCEV | Rat | Compression (location not reported) | IV (30 min + repeated) | reported | blinded | Strong (TEM, NTA, WB, EV markers TSG101, CD9, ALIX) | Mitophagy (AMPK axis) + multi-level analysis | Model heterogeneity |
| **Pan 2022** | SCEV | Rat (Wistar) | Contusion (T10) | IV, repeated | unclear | blinded (BBB) | Moderate-strong (TEM, NTA, WB) | Autophagy-mediated recovery | Randomization unclear |
| **Fan 2022** | OECEV | Rat (SD) | Crush (T8) | Local injection (immediate) | reported | unclear | Strong (TEM, NTA, WB; EV markers CD9, CD63) | Only SCI-specific OEC EV study; immune modulation | Single study; limited validation |
